# SOX9 maintains human foetal lung tip progenitor state by enhancing WNT and RTK signalling

**DOI:** 10.15252/embj.2022111338

**Published:** 2022-09-19

**Authors:** Dawei Sun, Oriol Llora Batlle, Jelle van den Ameele, John C Thomas, Peng He, Kyungtae Lim, Walfred Tang, Chufan Xu, Kerstin B Meyer, Sarah A Teichmann, John C Marioni, Stephen P Jackson, Andrea H Brand, Emma L Rawlins

**Affiliations:** ^1^ Wellcome Trust/CRUK Gurdon Institute University of Cambridge Cambridge UK; ^2^ Department of Physiology, Development and Neuroscience University of Cambridge Cambridge UK; ^3^ Department of Biochemistry University of Cambridge Cambridge UK; ^4^ Wellcome Sanger Institute Cambridge UK; ^5^ European Molecular Biology Laboratory European Bioinformatics Institute (EMBL‐EBI) Cambridge UK; ^6^ Department of Physics/Cavendish Laboratory University of Cambridge Cambridge UK; ^7^ Cancer Research UK Cambridge Institute University of Cambridge Cambridge UK; ^8^ Present address: Department of Clinical Neurosciences and MRC Mitochondrial Biology Unit University of Cambridge Cambridge UK; ^9^ Present address: Department of Anaesthesiology and Surgical Intensive Care Unit, Xinhua Hospital Shanghai Jiaotong University School of Medicine Shanghai China

**Keywords:** CRISPRi screen, ETVs, Lung organoids, SOX9, Targeted DamID, Chromatin, Transcription & Genomics, Development, Stem Cells & Regenerative Medicine

## Abstract

The balance between self‐renewal and differentiation in human foetal lung epithelial progenitors controls the size and function of the adult organ. Moreover, progenitor cell gene regulation networks are employed by both regenerating and malignant lung cells, where modulators of their effects could potentially be of therapeutic value. Details of the molecular networks controlling human lung progenitor self‐renewal remain unknown. We performed the first CRISPRi screen in primary human lung organoids to identify transcription factors controlling progenitor self‐renewal. We show that SOX9 promotes proliferation of lung progenitors and inhibits precocious airway differentiation. Moreover, by identifying direct transcriptional targets using Targeted DamID, we place SOX9 at the centre of a transcriptional network, which amplifies WNT and RTK signalling to stabilise the progenitor cell state. In addition, the proof‐of‐principle CRISPRi screen and Targeted DamID tools establish a new workflow for using primary human organoids to elucidate detailed functional mechanisms underlying normal development and disease.

## Graphical abstract

A CRISPRi screen and targeted [HV1] DamID in human lung organoids identifies SOX9 as the regulator of human lung progenitor cell self‐renewal.

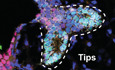

## Introduction

Respiratory disease is a leading cause of human mortality and morbidity worldwide (Labaki & Han, 2020). Multiple studies suggest that changes in human lung development can contribute to adult‐onset respiratory disease (Smith *et al*, 2018; Sakornsakolpat *et al*, 2019). Lung adenocarcinomas often take on an embryonic progenitor phenotype (Pacheco‐Pinedo *et al*, 2011; Laughney *et al*, 2020). Moreover, it is speculated that a long‐term cure for many respiratory diseases would be to trigger developmental processes in diseased lungs to regenerate the tissue (Morrisey & Hogan, 2010; Kotton & Morrisey, 2014; Nikolić *et al*, 2018). It is therefore crucial to understand the detailed molecular networks, which control human foetal lung progenitor self‐renewal and differentiation.

Mouse research has revealed that cells located at the distal tips of the branching lung epithelium are multipotent progenitors, which give rise to all lung epithelial lineages during development (Rawlins *et al*, 2009; Alanis *et al*, 2014). The cell signalling and transcription factor (TF) networks controlling mouse tip progenitors are increasingly well described (Okubo *et al*, 2005; Zhang *et al*, 2008; Chang *et al*, 2013; Rockich *et al*, 2013; Herriges *et al*, 2015; Ostrin *et al*, 2018; Gerner‐Mauro *et al*, 2020). Analogous tip progenitor cells with similar gene expression profiles and multipotent differentiation capacity are also found in the developing human lung (Nikolić *et al*, 2017; Danopoulos *et al*, 2018; Miller *et al*, 2018). Moreover, induced pluripotent stem cell (iPSC)‐based and human tissue‐derived 3D organoid culture systems have been established to recapitulate key features of the *in vivo* human tip progenitors (Chen *et al*, 2017; Nikolić *et al*, 2017; Miller *et al*, 2018). These human models require many of the signals known to function in mouse lung development, including WNT and FGFs (Nikolić *et al*, 2017; Miller *et al*, 2018). Human *in vitro* models provide new opportunities to elucidate the details of the molecular events, which control human lung development. However, accounting for the variable genetic background of primary human samples to reach a robust conclusion on biological questions remains challenging, particularly as little data are available on the extent of biological variability in these cells.

CRISPR genetic perturbation tools have transformed biology research (Jinek *et al*, 2012; Cho *et al*, 2013; Cong *et al*, 2013; Mali *et al*, 2013). Large‐scale functional genomic studies using genome‐wide screens in cancer cell lines have revealed previously unappreciated genetic regulators and accelerated the drug discovery process (Sanjana *et al*, 2014; Doench *et al*, 2016; Bowden *et al*, 2020). CRISPR screens have started to be applied to 3D organoid systems, which can more faithfully recapitulate *in vivo* biological processes (Planas‐Paz *et al*, 2019; Ringel *et al*, 2020; Murakami *et al*, 2021), but so far have required large cell numbers to overcome variability. CRISPR interference (CRISPRi) is particularly useful for the generation of homogenous knock‐downs to study essential gene function (Gilbert *et al*, 2013, 2014; Mandegar *et al*, 2016). CRISPRi screens have been established in cancer cell lines and iPSCs (Gilbert *et al*, 2014; Horlbeck *et al*, 2016; Tian *et al*, 2019, 2021), but not yet in organoid cultures.

We used a CRISPRi screen to probe systematically the function of 49 transcription factors (TFs) in human foetal lung tip progenitors. Our screen identified TFs that positively and negatively regulate tip progenitor self‐renewal, including SOX9. We show that SOX9 functions by both promoting proliferation and inhibiting differentiation. Moreover, by combining an inducible CRISPRi system with Targeted DamID (TaDa) (Southall *et al*, 2013; Marshall & Brand, 2015; Cheetham *et al*, 2018), we have identified direct SOX9 transcriptional targets. These include two receptor tyrosine kinase (RTK) signalling effectors, *ETV4* and *ETV5*, which we show work with SOX9 to coregulate the tip progenitor programme, and LGR5, which is known to enhance WNT signalling activity. These data place SOX9 at the intersection of WNT and RTK signalling in the developing human lungs. Our use of a state‐of‐the‐art CRISPRi screen and TaDa to study TF function in a human tissue‐derived organoid system will facilitate future functional studies of the coding and noncoding genome in organoid‐based research.

## Results

### A CRISPRi screen identified TFs that regulate lung progenitor self‐renewal

**Figure 1:**
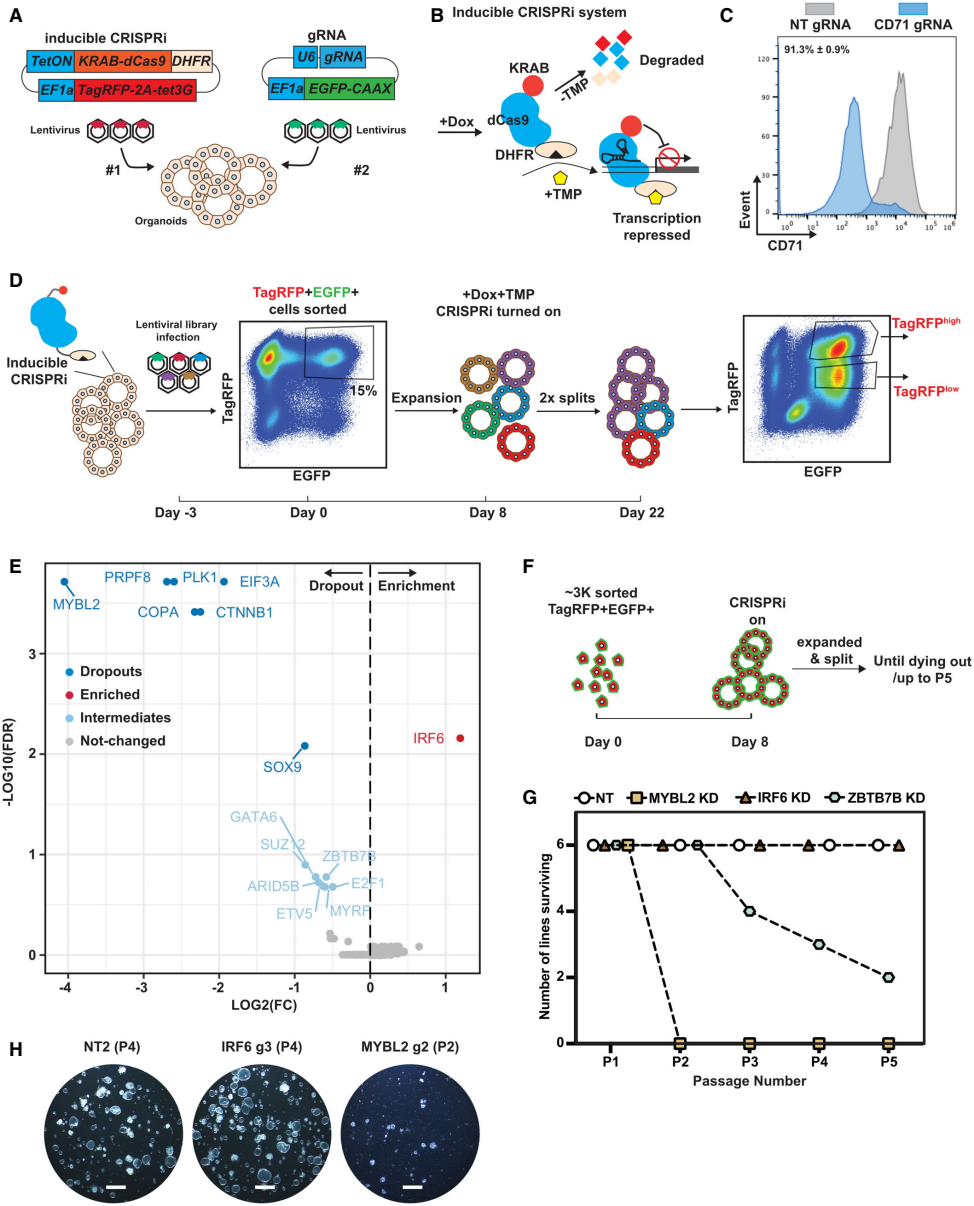
A CRISPRi screen identified crucial factors regulating human foetal lung progenitor cell self‐renewal Schematic of introducing inducible CRISPRi system into the organoid cells using serial lentiviral infection.Schematic of the inducible CRISPRi system. The inducible CRISPRi system was controlled by both Dox inducible system at mRNA level and TMP stabilisation of DHFR destabilising domain at protein level to achieve a tight control of CRISPRi function as previously reported (Sun *et al*, 2021).Representative flow cytometry results showing CD71, a cell surface marker, can be efficiently knocked‐down in the majority of organoid cells after 5 day of Dox and TMP treatment. *N* = 3 different organoid lines and 3 different gRNAs for CD71 were used. Mean ± SEM is labelled.Workflow for a focussed library CRISPRi screen for transcription factors regulating human foetal lung tip progenitor cell self‐renewal. Parental organoid lines with inducible CRISPRi system were established by lentiviral transduction and expanded. A CRISPRi gRNA library of 300 gRNAs was packaged into lentivirus and was used to infect single cells from two independent parental inducible CRISPRi organoid lines with an infection efficiency of ~ 15%. TagRFP^+^EGFP^+^ double positive organoid cells were collected. A fraction of cells was frozen for analysis of gRNA abundance in the starting population. The rest were seeded in Matrigel, given 8 day to recover and grown into small organoid colonies before treatment with Dox and TMP. Organoids were cultured in self‐renewing medium with Dox and TMP for 2 week before harvest. Organoids were physically broken into pieces for passaging twice during this period. TagRFP^high^EGFP^+^ and TagRFP^low^EGFP^+^ fractions were collected separately for downstream genomic DNA isolation and analysis.Volcano plot summarising gRNA abundance changes for each target gene. Strong depletion hits (−log10(FDR) > 2 and log2(FC) < 0) dark blue; strong enrichment hits (−log10(FDR) > 2 and log2(FC) > 0) red; intermediate depletion hits (1 < −log10(FDR) < 2 and log2(FC) < 0) light blue; unchanged genes grey. FDR, false discovery rate.Schematic of the experimental design for serial passaging assay to validate gene knock‐down effects on self‐renewal. ~ 3,000 TagRFP^high^EGFP^+^ double positive cells were harvested for each condition and seeded in Matrigel. Organoid cells were given 8 day to recover and grown into small colonies before treating with Dox and TMP. Organoids were then maintained in Dox and TMP and serially passaged by breaking into pieces every 3–4 day.Summary of serial passaging assay results for different knock‐downs. Three independent organoid lines each were transduced with two different gRNAs against the gene targets, making six organoid lines altogether.Representative wide field images to show organoid growth of the indicated gene knock‐down organoids at the indicated passage number. Scale bars denote 1 mm. Source data are available online for this figure.

To investigate systematically the TF network that regulates developing human lung epithelial tip progenitor cells, we performed a pooled CRISPRi screen in primary organoids. We used a tip progenitor cell organoid system, which faithfully recapitulates key markers and signalling pathways of the *in vivo* cells (Nikolić *et al*, 2017). Our knock‐down system (Sun *et al*, 2021) uses sequential lentiviral induction of an inducible CRISPRi vector, in which catalytically inactive Cas9 fused to the transcriptional repressor KRAB is controlled by doxycycline (Dox) and trimethoprim (TMP), and a constitutive gRNA (Fig 1A and B). After 4–5 day of drug treatment, targeted gene knock‐down can be achieved (Fig 1C).

**Figure EV1:**
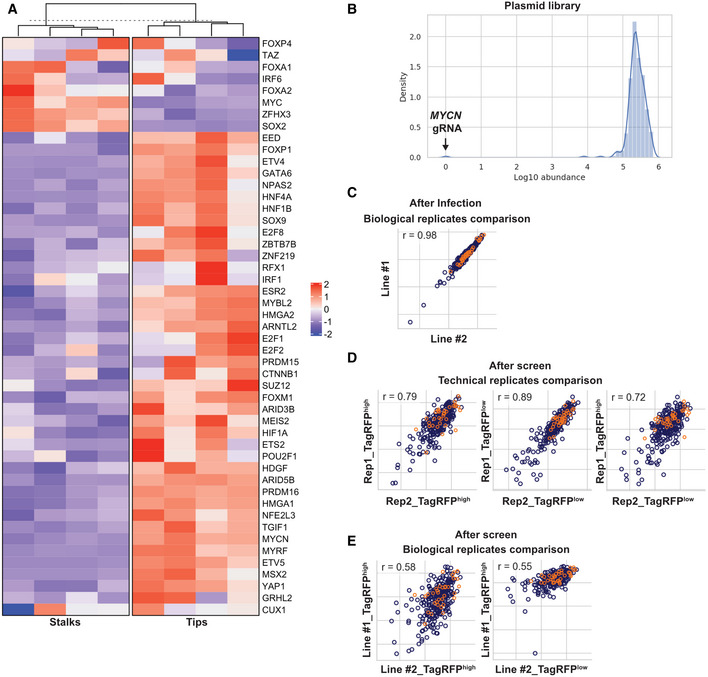
CRISPRi screen quality control Expression levels heatmap of the selected transcription factors in the developing human foetal lung tip progenitor and stalk cells. Data from Nikolić *et al* (2017).gRNA abundance distribution of the CRISPRi library after cloning into the plasmid vector. One gRNA targeting *MYCN* was missing; likely due to a gRNA synthesis issue.Pearson correlation of gRNA abundance between different samples indicated in axes. Between two independent CRISPRi parental lines 3 day after lentiviral transduction. *R* = 0.98 indicated great consistency of lentiviral transduction.Pearson correlation of gRNA abundance between technical replicates (Rep1 and Rep2). Great consistency was observed between TagRFP^high^ and TagRFP^low^ populations.Pearson correlation of gRNA abundance between biological replicates. A lower correlation was observed reflecting the variation of human tissue samples. Data information: Orange circles in (C–E) represent non‐targeting control gRNAs.

We designed a “drop‐out” screening strategy to identify TFs which promoted, or inhibited, human foetal lung tip progenitor self‐renewal (Fig 1D). We curated a lentiviral CRISPRi gRNA library containing on average 5 gRNAs per gene against 49 TFs exhibiting transcriptional enrichment and/or high abundance in tip progenitor cells (Fig EV1A; Dataset EV1), together with positive controls (4 essential genes) and negative controls (33 non‐targeting gRNAs), 300 gRNAs total. Library sequencing confirmed that all gRNAs were successfully cloned, except one gRNA against *MYCN* (Fig EV1B). Two independent parental organoid lines (biological replicates) with inducible CRISPRi were dissociated to single cells and transduced with the gRNA library at ~ 15% infection rate (Fig 1D), to ensure that most infected cells received one gRNA. At least 500 cells were infected per gRNA. TagRFP^+^EGFP^+^ double positive cells (containing both inducible CRISPRi and gRNA) were harvested 3 days after infection and we observed highly consistent gRNA abundance between transduced parental lines (biological replicates) (Fig EV1C). To test the robustness of the human foetal lung organoids for such screens, we also split the TagRFP^+^EGFP^+^ cells from one of the biological replicates and assayed them separately as technical replicates. Organoid cells were recovered for 8 days, then CRISPRi was switched on for 2 weeks of culture. Two different cell populations (TagRFP^high^EGFP^+^ and TagRFP^low^EGFP^+^) emerged at the end of the screen (Fig 1D). This was likely due to the inducible CRISPRi vector being modified by the cellular epigenetic machinery in long‐term cultures. We harvested TagRFP^high/low^ populations separately for downstream gRNA abundance analysis using next‐generation sequencing (NGS).

All expected gRNA sequences were present in all downstream NGS results (Dataset EV2). The consistency between the technical replicates (*R* = 0.79–0.89) demonstrated the feasibility of performing a pooled CRISPRi screen using ~ 500 cells per gRNA in the human foetal lung organoid system (Fig EV1D). Our gRNA correlation dropped to 0.57 when comparing different parental lines (biological replicates) (Fig EV1E), as might be expected due to the variable nature of organoids from different donors. The gRNA abundance of the TagRFP^high^EGFP^+^ versus TagRFP^low^EGFP^+^ populations correlated to a similar extent between technical (0.79 and 0.72) and biological (0.58 and 0.55) replicates (Fig EV1D and E). This suggested that these two populations behaved similarly, despite different TagRFP levels. Therefore, we treated them as technical replicates to find the most robust changes in gRNA abundance following passaging, “hits.”

gRNAs targeting all four positive‐control essential genes (*PRPF8*, *PLK1*, *COPA* and *EIF3A*) were strongly depleted in the screen (Fig 1E). Strong depletion of gRNAs targeting *MYBL2* and *CTNNB1* (Fig 1E) was consistent with *MYBL2* being a central factor regulating cell proliferation and the importance of WNT signalling for tip progenitor cell growth (Musa *et al*, 2017; Hein *et al*, 2022). gRNAs targeting *SOX9* were moderately depleted suggesting a role in tip progenitor self‐renewal, consistent with previous studies (Chang *et al*, 2013; Rockich *et al*, 2013; Li *et al*, 2021). By contrast, depletion of *SOX2* did not markedly influence progenitor self‐renewal, consistent with a recent knockout study (Sun *et al*, 2021). We also obtained intermediate hits, with gRNAs being mildly depleted, including *GATA6*, *SUZ12*, *ARID5B*, *ETV5*, *ZBTB7B*, *E2F1* and *MYRF*. Mild depletion could result from there being a few effective gRNAs buffered by the effects of nonfunctional gRNAs targeting the same gene; alternatively, non‐specific effects of a small group of gRNAs that influenced cell viability. Perhaps surprisingly, we discovered that gRNAs targeting *IRF6* were enriched in the screen, indicating a normal function in supressing self‐renewal, consistent with reports of a tumour suppressor role (Botti *et al*, 2011). These results indicate that the CRISPRi screen was able to effectively identify crucial factors regulating tip progenitor cell self‐renewal.

### Validation of CRISPRi hits confirmed screen robustness

**Figure EV2:**
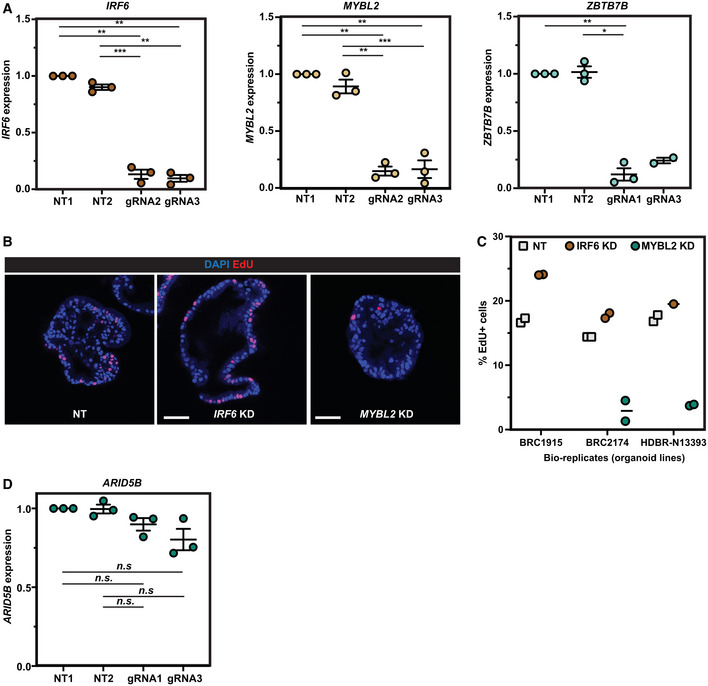
Validation of the CRISPRi screen results qRT–PCR results showing the targeted genes (*IRF6*, *MYBL2* and *ZBTB7B*) were efficiently knocked down by the inducible CRISPRi system using the gRNAs selected from the CRISPRi gRNA library.Representative EdU staining images of non‐targeting gRNA control and *IRF6* or *MYBL2* knock‐down experiments.Quantification of the percentage of EdU^+^ cells in each of three parental organoid lines used with non‐targeting control, *IRF6* knock‐down and *MYBL2* knock‐down. *n* = 1,649, 1,705, 3,548 cells were scored for NT controls. *n* = 2,517, 950, and 1,313 cells were scored for *IRF6* gRNAs. *n* = 1,098 and 1,306 cells were scored for *MYBL2* gRNAs.qRT–PCR results showing *ARID5B* was not knocked down by the inducible CRISPRi system using the gRNAs selected from the CRISPRi gRNA library. Data information: Error bars: mean ± SEM. Statistical analysis was using the two‐tailed paired *t*‐test. *P*‐values are reported as follows: *P < 0.05, **P < 0.01, ***P < 0.001 and n.s. non‐significant. *N* = 3 organoid lines (biological replicates) used for each panel. Source data are available online for this figure.

To validate the screen results, we cloned individual gRNAs separately into the gRNA vector and tested their effects on progenitor self‐renewal (Fig 1F). We selected *MYBL2*, *IRF6* and *ZBTB7B*/*ARID5B* as representative of strongly depleted, enriched and intermediately depleted genes, respectively. The gRNAs targeting *IRF6*, *MYBL2* and *ZBTB7B* were able to efficiently downregulate the expression of their targeted gene (Fig EV2A). We performed a serial passaging assay to test the effects of target gene knock‐down on organoid self‐renewal. *MYBL2* knock‐down organoids were rapidly lost after two serial passages in all the different organoid lines tested (6 of 6), whereas *ZBTB7B* knock‐down organoids were slowly lost in some of the organoid lines tested (4 of 6, by Passage 5) (Fig 1F–H). These data correlated well with the relative “strength” of hits in the screen. *IRF6* knock‐down led to better organoid growth compared with non‐targeting (NT) control organoids, consistent with its gRNA enrichment in the screen (Fig 1F–H). We further tested the effects of *IRF6* and *MYBL2* knock‐down on organoid proliferation using an EdU incorporation assay. *IRF6* knock‐down organoids exhibited a higher proportion of EdU^+^ cells compared with NT controls (Fig EV2B and C). Consistent with the serial passaging data, in *MYBL2* knock‐down organoids, the percentage of EdU^+^ cells was reduced (Fig EV2B and C). We did not observe any significant depletion of *ARID5B* mRNA following knock‐down (Fig EV2D), suggesting that the depletion of this gRNA in the screen might be due to off‐target effects. Overall, we were able to validate the strong, and some intermediate, hits in our screen confirming that the CRISPRi screening conditions were robust enough to discover TFs that regulate tip progenitor cell self‐renewal.

### SOX9 promotes proliferation and suppresses differentiation to govern progenitor self‐renewal

**Figure 2:**
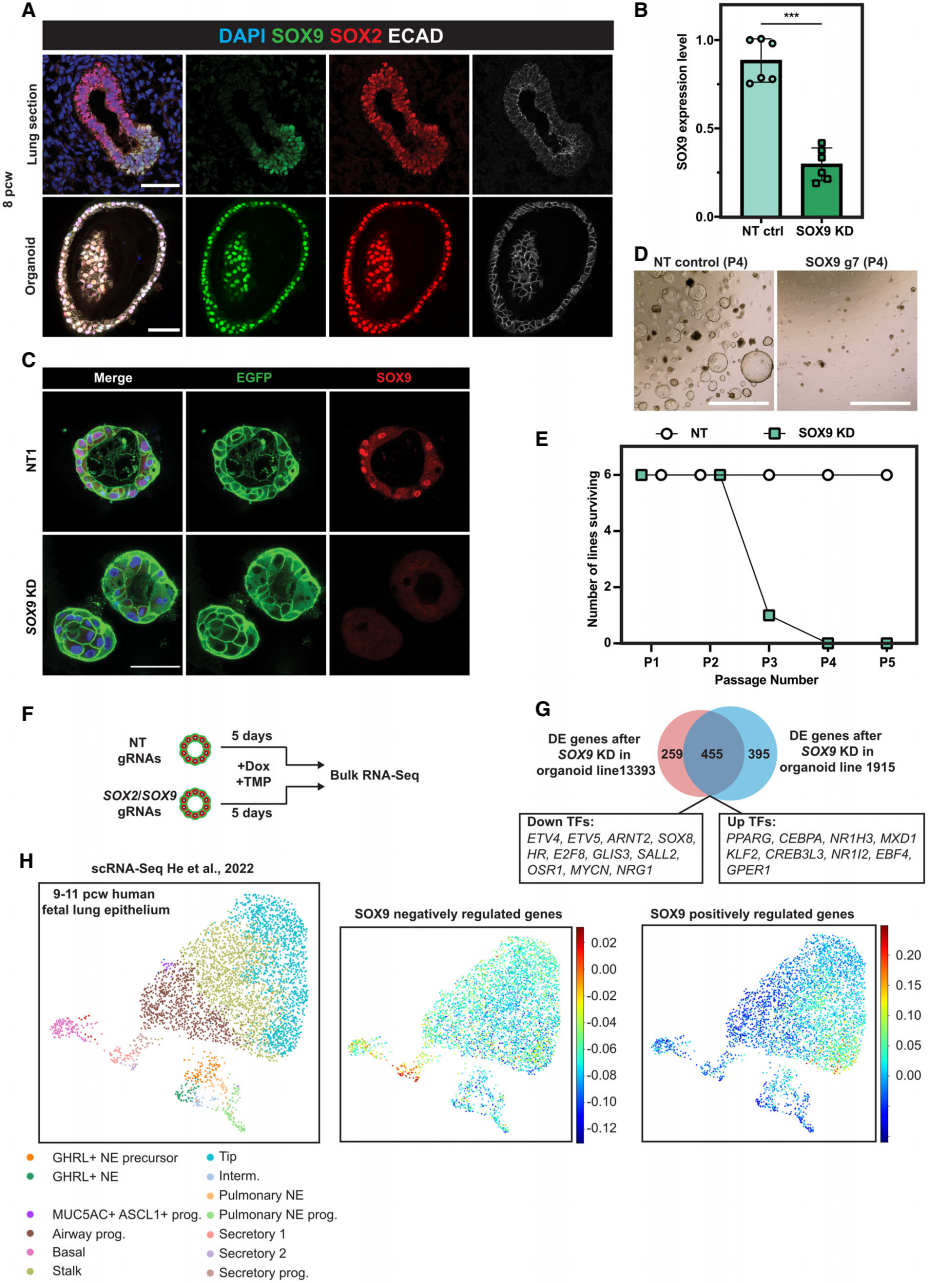
SOX9 regulates human foetal lung progenitor cell self‐renewal Human foetal lung tip organoids faithfully express *in vivo* tip progenitor markers, SOX2 and SOX9. Upper panel: SOX2/SOX9 dual expression in human foetal lungs. Lower panel: SOX2/SOX9 in human foetal lung tip organoids. SOX9 green, SOX2 red, E‐Cadherin white.qRT–PCR showing that *SOX9* gRNAs effectively knocked‐down *SOX9* transcript levels using inducible CRISPRi after 5 day of Dox and TMP treatment. Three different organoid lines and two different *SOX9* gRNAs were used. Error bars: mean ± SEM. Two‐sided Student's *t*‐test with equal variance ***P < 0.001.SOX9 was knocked down at the protein level using the inducible CRISPRi system after 4 day of Dox/TMP treatment.
*SOX9* knock‐down organoids at Passage #4 (P4).Summary of the serial passaging assay results for *SOX9* knock‐downs. Three independent organoid lines each were transduced with two different gRNAs against *SOX9*, making six different organoid lines altogether. For serial passaging assay, organoids were maintained in Dox and TMP and broken into pieces every 3–4 day.Schematic of RNA‐seq to identify SOX9 downstream targets. Two independent inducible CRISPRi parental organoid lines were transduced with 2 different non‐targeting control gRNAs, two different *SOX9* gRNAs and two different *SOX2* gRNAs, respectively. Organoids were supplemented with Dox/TMP for 5 day before harvesting for RNA‐seq.Venn Diagram showing the overlapping number of differentially expressed (DE) genes in *SOX9* knock‐down organoids from different parental lines.All SOX9 positively and negatively regulated genes were used to score against an scRNA‐Seq dataset from 9 to 11 pcw human foetal lung epithelium (left panel, preprint: He *et al*, 2022). SOX9 negatively regulated genes were primarily enriched in secretory lineage populations (middle panel). SOX9 positively regulated genes were primarily enriched in tip progenitor cells (right panel). Data information: Scale bars denote 50 μm (A, C) and 100 μm (D). Source data are available online for this figure.

SOX9 is an established lung epithelial tip progenitor marker. Moreover, *SOX9* knockouts in the developing mouse lung give phenotypes that are consistent with a progenitor self‐renewal defect, but the mechanism underlying the observed effects has not been elucidated (Chang *et al*, 2013; Rockich *et al*, 2013). In addition, although SOX9 has a reported phenotype in iPSC‐derived human lung cells, the molecular mechanisms underlying these effects are not clear (Li *et al*, 2021). We therefore used our human foetal lung progenitor organoids, which capture key TF expression patterns (Fig 2A), to explore the detailed mechanisms of *SOX9* function in multipotent lung progenitor cells. We reasoned that SOX proteins elicit diverse functions in distinct biological processes by changing their binding partners (Kamachi & Kondoh, 2013); therefore, the use of the self‐renewing organoids would provide a simple and isolated system to understand the cellular and molecular mechanisms of SOX9 in self‐renewal.

We first validated that the gRNAs used in the screen were able to knock‐down *SOX9* effectively (Fig 2B and C). *SOX9* knock‐down organoid cells were gradually lost in a serial passaging assay in all the organoid lines tested (6 of 6) (Fig 2D and E), confirming that SOX9 is an important regulator of tip progenitor cell self‐renewal.

We performed RNA‐seq on *SOX9* knock‐down organoids to characterise downstream targets. To account for biological variation between organoids from different donors and identify the most robust downstream targets we used the following: (i) two different gRNAs targeting *SOX9* to circumvent potential gRNA‐dependent non‐specific effects; (ii) two different NT control gRNAs to eliminate lentiviral transduction‐dependent non‐specific effects; (iii) two independent organoid lines to avoid possible organoid line non‐specific effects. *SOX2* knock‐down organoids were included for comparison. Organoids were harvested after 5‐day Dox/TMP treatment to probe the early effects of *SOX9* knock‐down (Fig 2F).

**Figure EV3:**
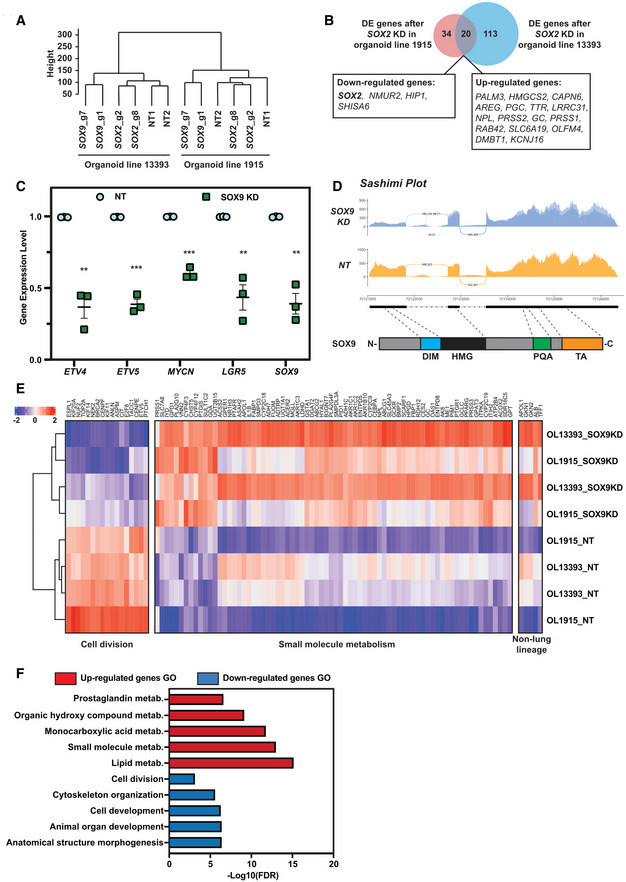
*SOX2* and *SOX9* knock‐down resulted in different transcriptome changes Unsupervised hierarchical clustering of non‐targeting control, *SOX2* knock‐down and *SOX9* knock‐down RNA‐Seq results.Venn diagram showing minimal overlap of differentially expressed genes after *SOX2* knock‐down in two different parental organoid lines. Overlapping DE genes were labelled in boxes.qPCR of selected DE genes from *SOX9* RNA‐seq data following *SOX9* knock‐down in a further 2 independent organoid lines. Cells harvested 5 days after knock‐down. Error bars: mean ± SEM. Statistical analysis was using the two‐tailed paired *t*‐test. *P*‐values are reported as follows: **P < 0.01, ***P < 0.001. *N* = 3 bio‐replicates (Organoid line BRC2174 with two different NT gRNAs and two different SOX9 gRNAs, and Organoid line BRC2136 with 1 NT gRNA and 1 SOX9 gRNA) were used.Sashimi plot to visualise splicing junction of NT control and *SOX9* KD. Upper panel: Sashimi plot was used to visualise splicing junction information in non‐targeting gRNA control and *SOX9* knock‐down groups. Junctional reads between intron #1 and exon #2 were only observed in *SOX9* knock‐down groups and not in non‐targeting gRNA control groups. Lower panel: major SOX9 domains in relation to the *SOX9* genomic locus. Exon #1 contains DIM and part of the HMG domain. DIM, dimerization domain. HMG, high‐mobility group domain. PQA, proline‐glutamine‐alanine repeats domain. TA, transactivation domain.Heatmap of gene expression from representative GO terms: cell division and small molecule metabolism together with gene expression of upregulated non‐lung lineage genes. OL, organoid line.Selected GO enrichment in DE genes after *SOX9* knock‐down. Source data are available online for this figure.

Unsupervised hierarchical clustering of the RNA‐seq transcriptome data revealed that the two independent organoid lines clustered separately (Fig EV3A), indicating that there was an organoid line‐dependent effect. However, within each cluster, NT controls and *SOX2* knock‐down organoids clustered separately from *SOX9* knock‐down organoids, suggesting that *SOX9* knock‐down led to unique gene expression profiles (Fig EV3A). To obtain the most robust differentially expressed (DE) genes, we first identified DE genes between the *SOX9*, or *SOX2*, knock‐down groups and NT controls of the same organoid line using a 2‐fold change in gene expression and an adjusted *P*‐value ≤ 0.05 as cut‐offs. We then overlapped the DE gene lists between biological replicates.

For *SOX2* knock‐down, this strategy identified 54 genes in organoid line 1915 and 133 genes in organoid line 13393 with an overlap of only 20 genes (including *SOX2*) (Fig EV3B; Dataset EV3), supporting our previous conclusion that SOX2 is dispensable for progenitor cell self‐renewal (Sun *et al*, 2021). By contrast, 850 genes (organoid line 1915) and 714 genes (organoid line 13393) were differentially expressed after *SOX9* knock‐down, with an overlap of 455 genes, including 20 TFs (Fig 2G; Dataset EV3). For *SOX9*, > 50% of the DE genes were shared between biological replicates (53.5% for line 1915 and 63.7% for line 13393) (Fig 2G), indicating that the experimental design was able to remove most non‐specific effects. To confirm that the DE gene list was robust, we repeated the *SOX9* knock‐down in a further two biologically independent organoid lines and confirmed the gene expression changes of a small number of these DE genes (Fig EV3C).

*SOX9* itself was not in the *SOX9* knock‐down DE gene list (Fig 2G), even though we had previously validated both mRNA and protein depletion (Fig 2B–C). We hypothesised that an alternative splicing event might be enhanced by CRISPRi‐induced knock‐down, buffering overall *SOX9* locus transcription. We therefore used sashimi plots to visualise the splice junctions (Fig EV3D). This showed that there was an alternative transcriptional start site(s) (TSS) within *SOX9* intron #1 and that hybrid reads of intron #1 and exon #2 emerged after *SOX9* knock‐down. However, the “new” *SOX9* transcript was unlikely to be functional as it lacked the dimerization domain and part of the HMG domain encoded in SOX9 exon #1 (Fig EV3D). Additionally, following knock‐down, we were unable to detect SOX9 using an antibody against the C‐terminus of the protein (Fig 2C).

To test whether the *SOX9* knock‐down cells altered their differentiation status, we took advantage of our human foetal lung scRNA‐seq dataset (preprint: He *et al*, 2022). We subset the human foetal lung scRNA‐seq data from 9 to 11 postconception weeks (pcw) and used all SOX9 positively and negatively regulated genes to score the epithelial compartment (Fig 2H). SOX9 negatively regulated genes were primarily expressed in secretory cells and their progenitors, indicating that SOX9 suppresses premature differentiation (Fig 2H, middle panel). By contrast, SOX9 positively regulated genes were primarily enriched in the tip progenitor cells, confirming that SOX9 functions to drive tip progenitor cell gene expression programmes (Fig 2H, right panel).

Gene ontology (GO) revealed that genes involved in cell metabolic processes were upregulated after *SOX9* knock‐down (Fig EV3E and F; Dataset EV4). Additionally, other foregut lineage markers, including from liver (*APOL1* and *ALB*) and stomach (*GKN1*, *GKN2* and *TFF1*), were upregulated (Fig EV3E). Cell division GO terms were enriched in the downregulated genes (Fig EV3F; Dataset EV5), consistent with the observation that *SOX9* knock‐down organoids were gradually lost in the serial passaging assay (Fig 2D and E). Overall, our results showed that SOX9 acts as a true self‐renewal factor in human lung tip progenitors by: (i) promoting tip cell proliferation; (ii) inhibiting differentiation by suppressing secretory cell lineage‐related metabolic genes and non‐lung lineage genes.

### Targeted DamID (TaDa) identified SOX9 direct transcriptional targets

**Figure 3:**
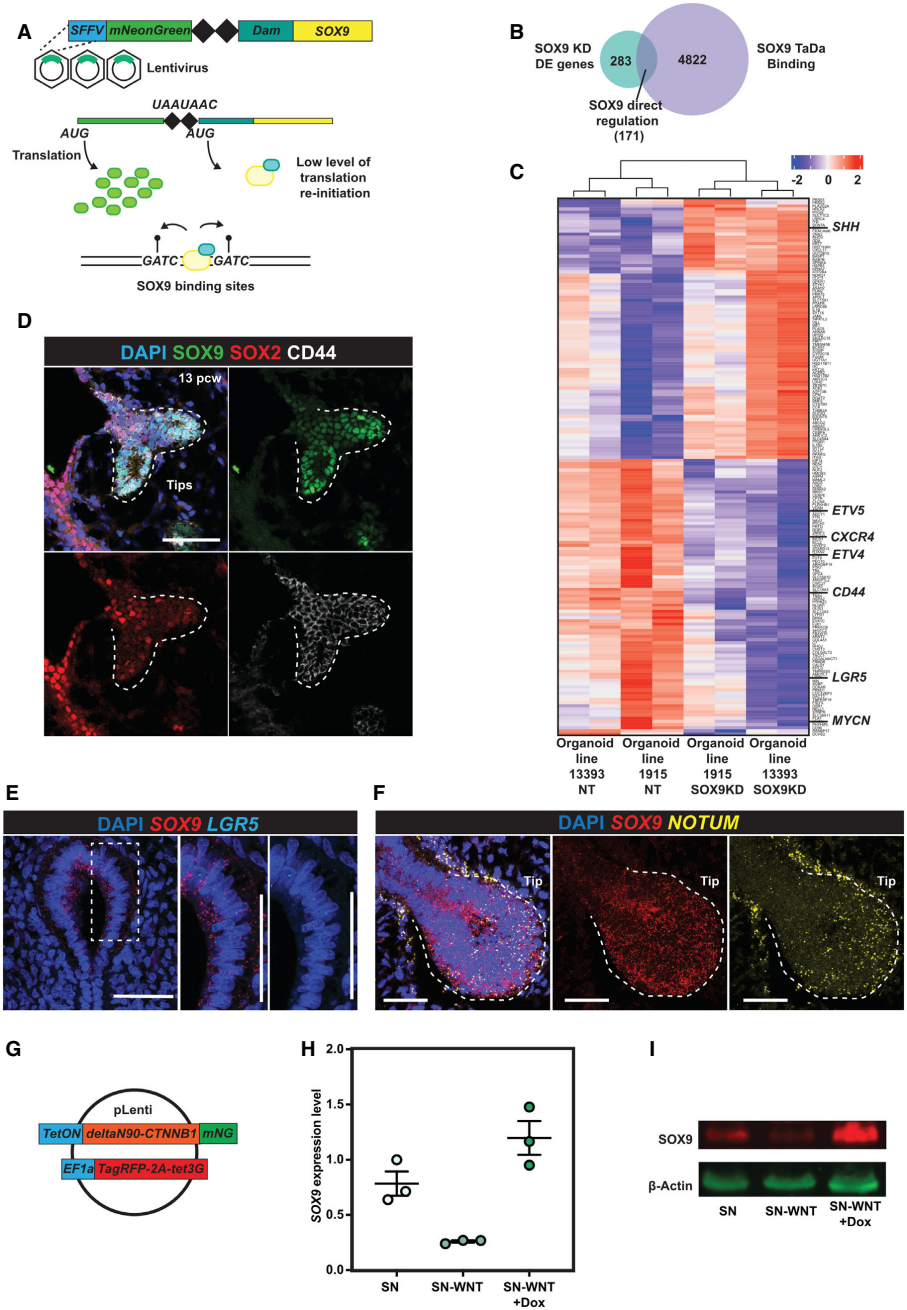
Targeted DamID (TaDa) identified SOX9 directly regulated targets Schematic of *SOX9* TaDa lentiviral construct design. SOX9 is fused with *E*.*coli* DNA adenine methylase (Dam). The fusion protein binds to SOX9 binding targets and methylates adenine in the sequence *GATC*. Methylated *GATC* is specifically recognised and cleaved by DpnI restriction enzyme. TaDa is designed to produce an extremely low level of Dam‐Sox9 fusion protein: an mNeonGreen open reading frame (ORF1) was placed in front of *Dam‐SOX9* fusion (ORF2). The two ORFs were separated by two stop codons and a frame‐shift C (represented by 2 black diamonds), such that the Dam‐SOX9 fusion protein is translated at very low levels after rare translational re‐initiation events.Venn Diagram showing the overlap between DE genes identified in the *SOX9* knock‐down RNA‐seq experiment and genes annotated from SOX9 TaDa peaks.Heatmap showing expression level of all 171 SOX9 directly regulated genes across nontargeting control and *SOX9* knock‐down organoid lines.CD44 protein expression in SOX9^+^ tip progenitor cells. SOX9 green, SOX2 red, CD44 white.
*LGR5* mRNA expression enrichment in *SOX9* expressing tip progenitor cells. *SOX9* red, *LGR5* cyan.Tip progenitor cells are of high WNT signalling activity. *NOTUM* yellow, *SOX9* red.Design of constitutively activated β‐catenin overexpression lentiviral construct.qRT‐PCR showing rescue of *SOX9* transcription after constitutively activated β‐catenin overexpression in organoids cultured without WNT activators. *N* = 3 different organoid lines. Error bars: mean ± SEM.WB showing SOX9 protein was rescued after constitutively activated β‐catenin overexpression in organoids cultured without WNT activators. Data information: Scale bars = 50 μm in all panels. Source data are available online for this figure.

**Figure EV4:**
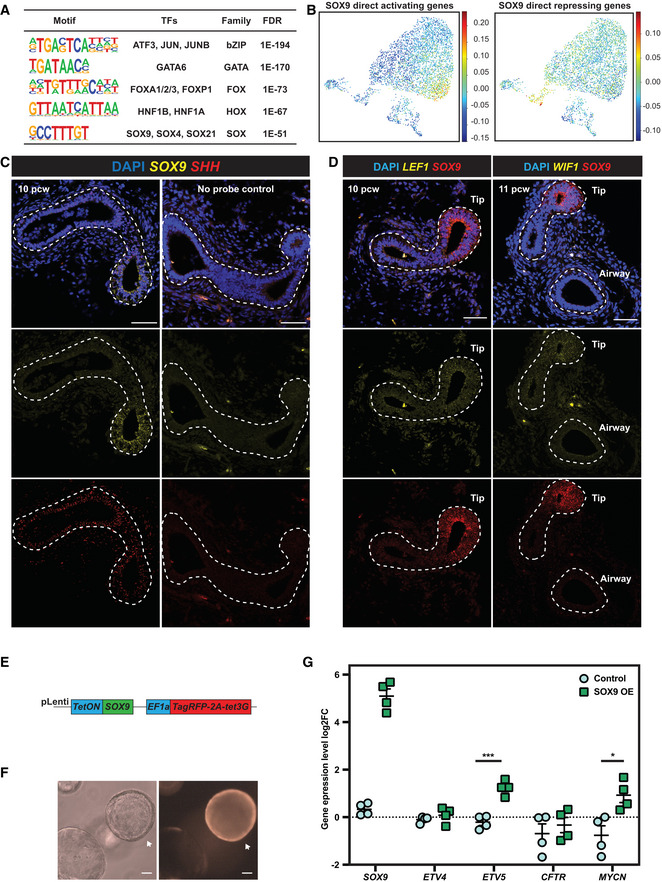
SOX9 directly activates tip cell genes and represses secretory cell genes Summary of enriched TF binding motifs in SOX9 TaDa peaks. The SOX motif was enriched, indicating the SOX9 TaDa faithfully identified SOX9 binding sites across the genome.SOX9 direct transcriptional target enrichment in human foetal lung scRNA‐seq data. All SOX9 direct transcriptional targets were used for scoring. Similar to Fig 2H, SOX9 directly activated targets were enriched in tip progenitor cells (left panel), whereas SOX9 directly repressed targets were enriched in secretory cell lineages (right panel).
*SHH* was co‐expressed with *SOX9* in human foetal lung tip progenitor cells. S*OX9* in yellow and *SHH* in red. No‐probe controls are shown in the right panel.
*LEF1* and *WIF1* were co‐expressed with *SOX9* in human foetal lung tip progenitor cells. S*OX9* in red and *LEF1* (left panel) and *WIF1* (right panel) in yellow.Lentiviral construct design for overexpressing SOX9 in human foetal lung progenitor cells.Representative images showing organoid morphology does not change after 3 days of SOX9 overexpression. SOX9 overexpressed organoid indicated with arrow.qRT–PCR results showing that 3 days of SOX9 overexpression led to *ETV5* and *MYCN* transcription being significantly upregulated, however, *ETV4* and *CFTR* were not changed. *N* = 4 organoid lines (bio‐replicates) were used. Error bars: mean ± SEM. Two‐tailed Student's *t*‐tests were performed. *P*‐values are reported as follows: *P < 0.05; ***P < 0.001. Data information: Scale bars denote 50 μm (C, D) and 100 μm (F). Source data are available online for this figure.

To identify genes directly activated or repressed by SOX9, we used SOX9 Targeted DamID (TaDa) to probe genomic occupancy (Southall *et al*, 2013; Marshall & Brand, 2015; Marshall *et al*, 2016; Cheetham *et al*, 2018). We introduced the TaDa system into organoids via lentivirus and harvested cells 48–72 h after transduction (Fig 3A). 4845 SOX9 binding peaks were called with high confidence (FDR < 10^−50^) versus Dam‐only controls (no SOX9 fusion) and shared by four independent organoid lines (Dataset EV6). *De novo* motif analysis revealed the enrichment of a SOX motif (Fig EV4A), suggesting that the SOX9‐Dam fusion protein faithfully bound to SOX9 targets. We overlapped the *SOX9* knock‐down DE gene list with peak annotations (http://great.stanford.edu/public/html/, GREAT analysis) and identified 171 genes directly regulated by SOX9 (Fig 3B; Dataset EV3). SOX9 positively regulated genes were enriched in tip progenitor cells and negatively regulated genes enriched in secretory cell lineages (Fig EV4B). Direct transcriptional targets included *ETV4*, *ETV5*, *MYCN*, *LGR5*, *ROR1*, *CD44*, *CXCR4* and *SHH*, many of which have been previously reported to be important for lung development in mouse (Fig 3C) (Weaver *et al*, 2003; Okubo *et al*, 2005; Herriges *et al*, 2015; Hein *et al*, 2022). *SHH* expression in human tip progenitor cells was confirmed by *in situ* HCR (hybridization chain reaction) (Fig EV4C).

Human lung bud tip progenitors are dependent on WNT activation for their *in vitro* growth (Nikolić *et al*, 2017; Miller *et al*, 2018). Confirming this, *CTNNB1* encoding β‐catenin, an intracellular signal transducer of the WNT signalling pathway, was a strong hit in our screen (Fig 1E). We confirmed that CD44 protein (Fig 3D) and *LGR5* mRNA (Fig 3E) localised in *SOX9*‐expressing tip regions, consistent with these genes being direct SOX9‐targets and with previous reports of *LGR5* expression (Ostrin *et al*, 2018; Hein *et al*, 2022). LGR5 and CD44 are WNT signalling amplifiers (Schuijers & Clevers, 2012; Schmitt *et al*, 2015), suggesting that SOX9 could enhance WNT signalling in tip progenitors. We also observed that known WNT targets and feedback inhibitors including, *NOTUM* (Gerhardt *et al*, 2018), *LEF1* (Gerner‐Mauro *et al*, 2020) and *WIF1* (Hsieh *et al*, 1999) are enriched in human lung bud tip progenitors (Figs 3F and EV4D) confirming that human lung bud tips are highly WNT responsive. This prompted us to investigate whether WNT signalling influenced *SOX9* expression. We removed WNT signalling activators (R‐spondin1 and CHIR99021) from tip progenitor organoid culture for 6 days, leading to loss of SOX9 transcript and protein (Fig 3H and I). We further showed that the regulation is via β‐catenin, canonical WNT signalling, by overexpressing a constitutively activated β‐catenin (DeltaN90‐β‐catenin) (Guo *et al*, 2012) in the absence of WNT signalling activators (Fig 3G), rescuing SOX9 expression (Fig 3H and I). Therefore, SOX9 is regulated by canonical WNT signalling and itself directly regulates *LGR5* and *CD44*, which are likely to further enhance WNT signalling. This is consistent with a recent report showing that WNT signalling is amplified by RSPO2‐LGR5 in the distal lung region and is required for human lung bud tip maintenance and SOX9 expression (Hein *et al*, 2022).

We used SOX9 overexpression in tip progenitor organoids to test some of the predicted direct transcriptional targets (Fig EV4E–G). *ETV5* and *MYCN* were significantly upregulated upon SOX9 overexpression, further supporting the direct regulation (Fig EV4G). However, *ETV4* and *CFTR* remained unchanged, likely due to already saturated binding at these loci (Fig EV4G). These results marked the first application of TaDa in organoids to identify TF binding targets.

### SOX9 and ETVs coordinate the tip progenitor self‐renewal programme

**Figure 4:**
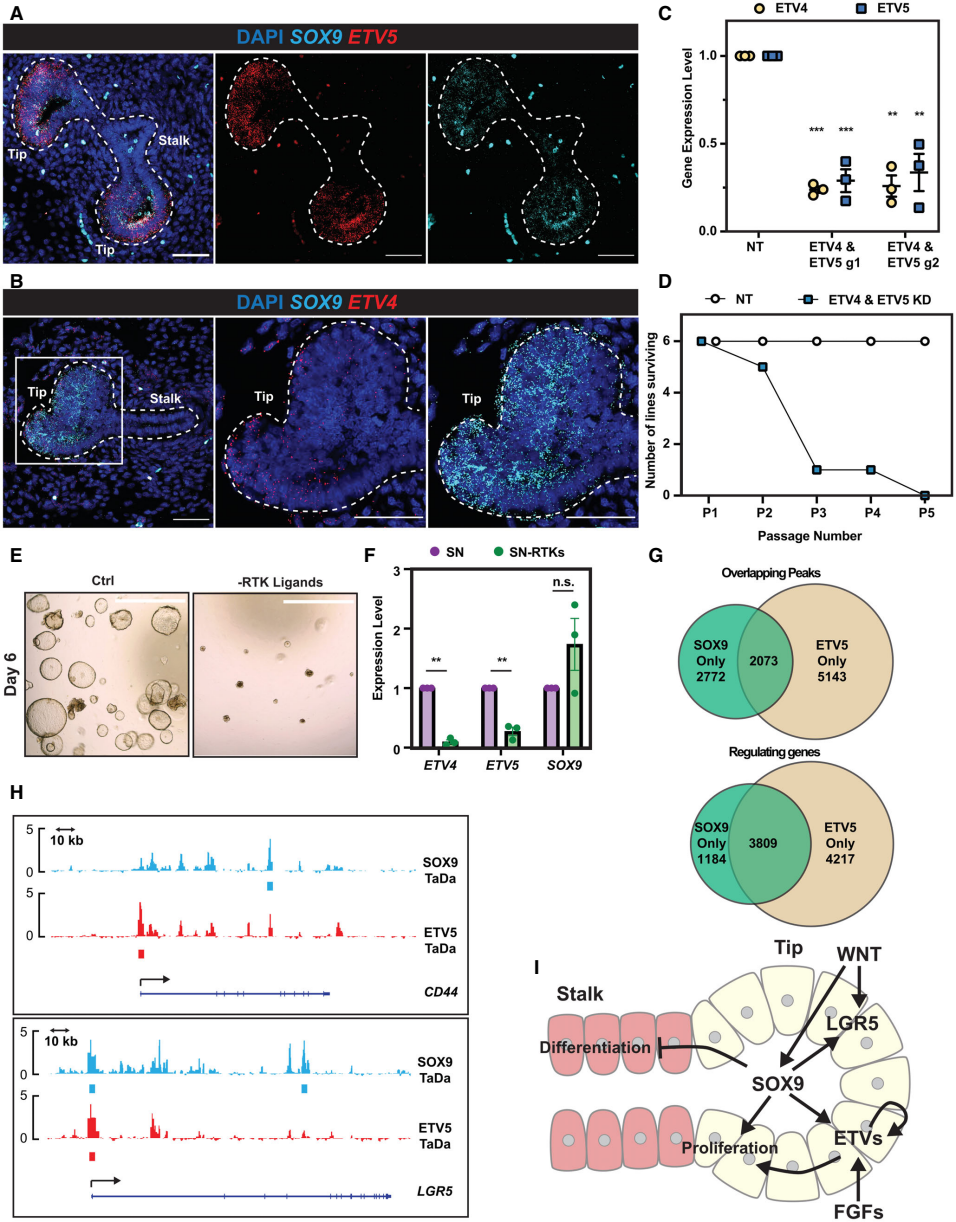
SOX9 directly regulates *ETV4* and *ETV5* expression, thereby enhancing FGF signalling A, BHCR images showing *ETV5* (A) and *ETV4* (B) expression in *SOX9* expressing tip progenitors. *ETV4/5* red, *SOX9* cyan.CqRT–PCR showing *ETV4* and *ETV5* dual knock‐down in tip organoids. *N* = 3 different organoid lines and 2 different *ETV4* and *ETV5* gRNA combinations were used. Error bars: mean ± SEM. Two‐tailed paired *t*‐test: **P < 0.01; ***P < 0.001.DSummary of the serial passaging assay results for *ETV4*; *ETV5* dual knock‐downs. Three independent organoid lines each were transduced with 2 different *ETV4*; *ETV5* gRNA combinations, making 6 different organoid lines altogether. Organoid cells were given 8 days to recover after plating and grown into small colonies before treating with Dox and TMP. Organoids were then maintained in Dox and TMP and serially passaged by breaking into pieces every 3–4 day.ERepresentative images of organoids grown in self‐renewing medium (left) and self‐renewing medium without RTK ligands (EGF, FGF7 and FGF10) (right).FqRT–PCR of *ETV4*, *ETV5* and *SOX9* expression level in organoids in self‐renewing medium and self‐renewing medium without RTK ligands. *N* = 3 different organoid lines used. Error bars: mean ± SEM. Two‐tailed paired *T*‐test: **P < 0.01; n.s. nonsignificant.GVenn diagrams showing overlap of SOX9‐ETV5 TaDa peak regions (upper) and overlapping genes from TaDa peak annotations (lower).HExamples of SOX9 and ETV5 genomic occupancies showing co‐regulation. At the *CD44* locus (upper), SOX9 binds to an intron, whereas ETV5 primarily occupied the promoter. By contrast, for the *LGR5* gene (lower), SOX9 and ETV5 both showed occupancy at the promoter.ISchematic model of SOX9 regulation of tip progenitor cell self‐renewal. Data information: Scale bars denote 50 μm (A, B) and 100 μm (E). Source data are available online for this figure.

We were intrigued to discover that *ETV4* and *ETV5* are SOX9 direct binding targets. We showed that *ETV5* and *ETV4* are enriched in *SOX9* expressing tip progenitor cells *in vivo* (Fig 4A and B). *ETV4* and *ETV5* are redundant in mouse lung development (Herriges *et al*, 2015). This is consistent with our screen results in which *ETV5* was an intermediate hit and *ETV4* gRNAs had no effect (Fig 1E). To test whether *ETV4* and *ETV5* function with SOX9 in human foetal lung progenitor self‐renewal, we knocked down *ETV4* and *ETV5* simultaneously using inducible CRISPRi (Fig 4C). *ETV4*; *ETV5* double knock‐down organoids were gradually lost during serial passaging, similar to *SOX9* knock‐down (Fig 4D).

**Figure EV5:**
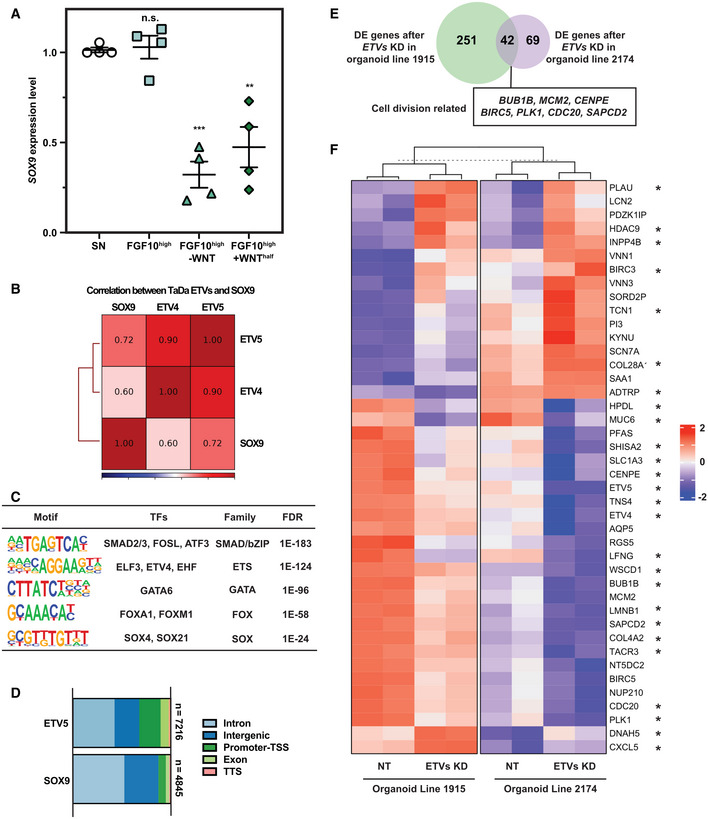
Identification of direct ETV5 binding targets qRT–PCR showing *SOX9* transcription 6 day after FGF10 supplementation (500 ng/ml), or FGF10 supplementation and removal of WNT activators. *N* = 4 different organoid lines. Error bars: mean ± SEM. Statistical analysis was using the two‐tailed paired *t*‐test. *P*‐values are reported as follows: **P < 0.01; ***P < 0.001; n.s. nonsignificant.Pearson correlation of SOX9, ETV4 and ETV5 TaDa. ETV4 and ETV5 TaDa exhibited great consistency.Motifs enriched in ETV5 TaDa peaks. The ETS binding motif was highly enriched.Genomic occupancy annotated features for SOX9 and ETV5 peaks.Venn diagram showing overlap of differentially expressed genes after *ETV4* and *ETV5* double knock‐down in two different parental organoid lines. Overlapping DE genes related to cell division by GO analysis are labelled in the box.Heatmap showing expression level of all 42 DE genes after *ETV4*; *ETV5* double knock‐down across different organoid lines. Directly regulated genes are marked by asterisks. Source data are available online for this figure.

ETVs are RTK signalling effectors (Zhang *et al*, 2009; Herriges *et al*, 2015) and RTK ligands are required for long‐term tip organoid self‐renewal (Nikolić *et al*, 2017). We reasoned that SOX9 influences RTK signalling, thereby influencing progenitor self‐renewal. Therefore, we tested the effects of removing RTK ligands (EGF, FGF10 and FGF7) from our self‐renewal medium; both *ETV4* and *ETV5* were down‐regulated and organoids could not proliferate normally (Fig 4E and F). *SOX9* transcription was not affected by 6 days of RTK ligand removal, suggesting its transcription is not directly controlled by RTK signalling (Fig 4F), even when high levels of FGF10 were supplied in combination with a reduction in WNT activation (Fig EV5A). These results show that SOX9 can directly activate *ETV4* and *ETV5* expression, enhancing RTK signalling.

To identify ETV4 and ETV5 binding targets, we performed TaDa. ETV4 and ETV5 TaDa exhibited > 90% similarity (Fig EV5B), and we primarily used the ETV5 data for analysis. Motif analysis on ETV5 bound regions identified the ETS binding motif, suggesting ETV5 TaDa recapitulated ETV5 binding events (Fig EV5C). The SOX motif was also mildly enriched in ETV5 binding regions. We explored the possibility of SOX9 and ETV5 coregulation of targets. Both SOX9 and ETV5 primarily bind to intronic regions (Fig EV5D), consistent with previous results (Shih *et al*, 2015). SOX9 and ETV5 binding peaks overlapped < 50% (SOX9: 42.8%; ETV5: 28.7%). However, peak annotation by GREAT analysis revealed a much greater overlap (SOX9: 76.3%; ETV5: 47.5%) (Fig 4G), suggesting SOX9 and ETV5 bind to different regions of the same loci to coordinate the tip progenitor self‐renewal programme (Fig 4H).

To identify gene expression changes following *ETV4*; *ETV5* double knock‐down, we performed RNA‐seq. Using a less‐stringent cut‐off (1.5‐fold change and FDR ≤ 0.05), *ETV4*; *ETV5* double knock‐down resulted in many fewer gene expression changes when compared with *SOX9* knock‐down (Fig EV5E, compare with Fig 2G). Only 42 differentially expressed genes were identified that were common to both *ETV4*; *ETV5* double knock‐down organoid lines and both sets of gRNAs; these were predominantly related to cell proliferation (Fig EV5E and F; Dataset EV7). Of the 42 differentially expressed genes, 26 were bound by ETV5 in the TaDa assay (Fig EV5F; Dataset EV7) suggesting direct regulation. Only six of these were also SOX9 direct regulated targets (*CENPE*, *ETV4*, *ETV5*, *RGS5*, *SHISA2 and TNS4*). Taken together, the ETV5 TaDa binding data and *ETV4*; *ETV5* double knock‐down DE genes show that ETV4 and ETV5 are binding widely throughout the genome of lung bud tip progenitors, but on their own are only activating or repressing transcription to a detectable level at a small number of loci (mostly concerned with cell proliferation). This is analogous to other studies where ETV5 binding has been compared with transcriptional changes following its depletion (Zhang *et al*, 2017; Kalkan *et al*, 2019) and suggests that it mostly works cooperatively with other TFs, such as SOX9, at the majority of its binding sites.

Our results suggest a model for SOX9 function in human foetal lung progenitors (Fig 4I), in which it promotes self‐renewal by: (i) promoting progenitor cell proliferation, via a feed‐forward WNT signalling loop and by enhancing RTK signalling via ETV factors; (ii) suppressing metabolic processes favourable for precocious differentiation in early lung development; (iii) suppressing other non‐lung lineage gene expression to maintain human foetal lung progenitor states.

## Discussion

CRISPR‐related screens have rarely been applied to organoids because of the difficulties in manipulating 3D culture systems. We have performed the first pooled CRISPRi dropout screen in a tissue‐derived organoid system, followed by detailed characterisation of selected gene function. Previous reports challenged the robustness of CRISPR screens in organoid systems (Ringel *et al*, 2020), or used an enormous cell number (10,000 cells per guide) (Planas‐Paz *et al*, 2019). Here, we achieved a pooled CRISPRi screen of high robustness and sensitivity with a starting population of ~ 500 cells per gRNA (Figs 1 and EV1 and EV2). Our ~ 500 cell representation per gRNA is similar to screens carried out in cancer cell lines (Joung *et al*, 2017; Bowden *et al*, 2020). We envision that similar screens can be practically and affordably scaled‐up for targeting hundreds of genes, for example the mammalian TFome (Ng *et al*, 2021). Moreover, the future use of functional genomics, including CRISPR and compound library screening, for modifiers of respiratory disease‐relevant phenotypes in human organoid models will be a powerful clinical research tool. Such screens could be performed in pooled or arrayed format. They could assay cell survival/proliferation as in this work, or specific phenotypes such as protein expression or localisation, or be combined with Perturb‐seq (Dixit *et al*, 2016; Datlinger *et al*, 2017) to directly assay cell states.

There is great potential for clinical research based on respiratory organoid functional genomics. However, it is important to be aware of the limitations of such screens. We chose to use CRISPRi to modulate gene expression because we had a tightly inducible system (Sun *et al*, 2021), human CRISPRi gRNA libraries were available (Horlbeck *et al*, 2016), and we find that gene expression is very homogenous within a population of knocked‐down cells (Fig 1C). No genetic manipulation system is perfect, and even within this small‐scale screen, we found genes that we could not knock down (*ARID5B*), had cryptic transcriptional start sites following knock‐down (*SOX9*), or were highly redundant (*ETV4* and *ETV5*). It is likely that similar limitations would have been encountered with any CRISPR system. It is therefore important to be aware that any screen will have false‐positives and negatives and require detailed mechanistic follow‐up. Similarly, human organoid systems are proving to be important models of disease, but they are reductionist models and effective selection of disease‐relevant phenotypes will be crucial for their future impact in clinical research. Finally, we found that within the screen, there was variability between organoid lines (Fig EV1E; *r* = 0.55, 0.58), introducing additional complexity compared with screens performed on mouse cells or immortalised cell lines. However, this genetic background variability could also be viewed as introducing the potential to couple functional genomics with human genetics for the development of future precision medicine therapies.

During this study, we performed RNA‐seq on primary human tissue‐derived organoids following knock‐down of *SOX2*, *SOX9* and *ETV4/5*. Each time we observed a significant level of transcriptome‐level variability between biological replicates, even though the cellular level phenotypes observed were robust across multiple organoid lines. This transcriptome‐level variability is a further challenge of working with primary human samples that is rarely discussed. In our NGS experiments, we chose to perform controls for lentiviral infection (2 non‐targeting gRNAs per experiment), gRNA effectiveness (2 targeting gRNAs per experiment) and biological variation (2 independent biological replicates per experiment) and to focus on differentially expressed genes that were identified between all conditions; a pragmatic approach between affordability and replication. However, it is possible that this strategy will under‐represent transcriptional changes when the fold‐changes are low, as potentially observed in the *ETV4/5* double knock‐down.

Our study is the first to use TaDa in human organoids (Figs 3 and 4). TaDa is independent of ChIP‐grade antibodies and requires a relatively low cell number (400 K cells for TaDa in this study, typically > 1 million cells for ChIP‐Seq) (Southall *et al*, 2013; Cheetham *et al*, 2018; Tosti *et al*, 2018). We discovered *ETV4* and *ETV5* (Fig 3C), among others, as SOX9 directly regulated targets. Interestingly, such regulation was not discovered in *SOX9* knockout mouse lungs. This could be due to gene expression changes being masked by non‐SOX9 expressing cells in the previous mouse study (Chang *et al*, 2013), or species‐specific roles. We demonstrate that SOX9 promotes FGF signalling by activating *ETV4/5* transcription (Fig 3). However, in the short‐term culture experiments we have performed, FGF ligands (Fig 4F) and ETV4/5 DNA binding (Fig EV5) are not required to activate *SOX9* transcription, placing SOX9 “higher” in the self‐renewal network. This is consistent with a mouse study of *ETV4/5* knockout, which did not lead to SOX9 expression pattern changes (Herriges *et al*, 2015). Additionally, in support of an upstream role for SOX9, our analysis of gene expression changes following knock‐down shows that SOX9 both promotes proliferation and represses differentiation, whereas ETV4/5 promote proliferation only.

It is of great interest that ETV4/5 bind widely in the genome and are predicted to coregulate many genes with SOX9 (Fig 4G). Moreover, binding site analysis of both the SOX9 and the ETV4/5 TaDa data identified motifs for other TFs that are known to be expressed in the tip progenitors (Figs EV4A and EV5A), including FOXA, GATA and HNF1 families. These data suggest that a highly redundant network of TFs may regulate the tip progenitor self‐renewal gene regulation network (GRN) and that future combinatorial manipulation of TF expression, combined with binding site analysis, will be required to determine their effects.

We have shown that WNT signalling is highly active in tip progenitor cells, that SOX9 directly regulates WNT pathway activators and that SOX9 expression is controlled by canonical WNT signalling (Fig 3C–I). These findings were consistent with previous mouse research showing that β‐catenin knockout led to SOX9 loss in tip progenitors (Ostrin *et al*, 2018) and also with the finding that blocking the LGR5‐WNT signalling axis in human foetal lung explants reduced the number of SOX9‐positive tip cells (Hein *et al*, 2022). Depletion of *SOX9* also results in an increased expression of gastrointestinal tract markers, some of which are direct SOX9 binding targets (*APOL1*, *TFF1*; Fig 3C). In mouse studies, both a reduction (Ostrin *et al*, 2018) and an increase (Okubo *et al*, 2005) in WNT signalling have been associated with the acquisition of gastrointestinal transcriptional phenotypes in the developing lung. However, our lung organoids were grown in the presence of the strong WNT activator, CHIR99021, making it difficult to determine whether WNT is also contributing to gastrointestinal gene expression in our experiments.

We have made the first effort to systematically identify transcription factors with roles in human lung development. These approaches and results will deepen our understanding of human lung development and disease, and greatly benefit future functional organoid‐based studies of the coding and non‐coding genome.

## Materials and Methods

### Human embryonic and foetal lung tissue

The sources of human foetal lung tissues were the MRC/Wellcome Trust Human Developmental Biology Resource (www.hdbr.org; London site REC reference: 18/LO/0822; Newcastle site REC reference: 18/NE/0290; Project 200454) and Cambridge University Hospitals NHS Foundation Trust under NHS Research Ethical Committee permission (96/085). None of the samples used for the current study had any known genetic abnormalities.

### Derivation and maintenance of human foetal lung organoid culture

Human foetal lung organoids were derived and maintained as previously reported (Nikolić *et al*, 2017). Briefly, human foetal lung tissues were dissociated with Dispase (8 U/ml Thermo Fisher Scientific, 17105041) at room temperature (RT) for 2 min. Mesenchyme was carefully removed by needles. Branching epithelial tips were micro‐dissected, transferred into 50 μl of Matrigel (356231, Corning) and seeded in 24‐well low‐attachment plate wells (M9312‐100EA, Greiner). The plate was incubated at 37°C for 5 min to solidify the Matrigel. 600 μl of self‐renewing medium containing: N2 (1: 100, ThermoFisher Scientific, 17502‐048), B27 (1: 50, ThermoFisher Scientific, 12587‐010), N‐acetylcysteine (1.25 mM, Merck, A9165), EGF (50 ng/ml, PeproTech, AF‐100‐15), FGF10 (100 ng/ml, PeproTech, 100–26), FGF7 (100 ng/ml, PeproTech, 100–19), Noggin (100 ng/ml, PeproTech, 120‐10C), R‐spondin (5% v/v, Stem Cell Institute, University of Cambridge), CHIR99021 (3 μM, Stem Cell Institute, University of Cambridge) and SB 431542 (10 μM, bio‐techne, 1614), was added. The plate was incubated under standard tissue culture conditions (37°C, 5% CO_2_). Once formed, organoids were maintained in self‐renewing medium and passaged for routine maintenance by mechanically breaking using P200 pipettes every 3–7 days.

### Whole mount immunostaining for human foetal lung organoids

Organoids were fixed with 4% paraformaldehyde (PFA) in the culture plates on ice for 30 min. After two PBS washes, 0.5% (w/v) bovine serum albumin (BSA), 0.2% Triton‐X in PBS (washing solution) was added and left on ice overnight to dissolve Matrigel. Organoids were then transferred into multiple CellCarrier‐96 Ultra Microplates (PerkinElmer, 6055300) for staining. Blocking was performed in washing solution with 5% donkey serum (Stratech, 017–000‐121‐JIR), 0.5% (w/v) bovine serum albumin (BSA) (blocking solution) at 4°C overnight. For primary antibody staining, the following antibodies in blocking solution were used at 4°C for 24–48 h: SOX2 (1: 500, Bio‐techne, AF2018), SOX9 (1: 500, Sigma, AB5535), E‐cadherin (1: 1,500, Thermo Fisher Scientific, 13‐1900), GFP (1: 500, AbCam, ab13970). After washing off the primary antibodies, the following secondary antibodies in washing buffer were used at 4°C overnight: donkey anti‐chick Alexa 488 (1: 2,000, Jackson Immune, 703‐545‐155), donkey anti‐rabbit Alexa 488 (1: 2,000, Thermo Fisher Scientific, A21206), donkey anti‐rabbit Alexa 594 (1: 2,000, Thermo Fisher Scientific, A‐21207), donkey anti‐goat Alexa 594 (1: 2,000, Thermo Fisher Scientific, A‐11058), donkey anti‐rat Alexa 647 (1: 2,000, Jackson Immune, 712‐605‐153). The following day, DAPI (Sigma, D9542) staining was performed in washing solution at 4°C for 30 min. After two washes with PBS, 97% (v/v) 2′‐2′‐thio‐diethanol (TDE, Sigma, 166782) in PBS was used for mounting. Confocal z stacks were acquired using Leica SP8 at an optical resolution of 1,024 × 1,024 using a 40× lens. Images were processed using ImageJ (version 2.0.0).

### Immunofluorescence for human foetal lung sections

Human foetal lung sections were rinsed PBS twice and were permeabilized with 0.3% Triton‐X in PBS for 10 min. After three PBS washes were carried out, 5% donkey serum (Stratech, 017–000‐121‐JIR), 0.5% (w/v) bovine serum albumin (BSA), 0.1% Triton‐X in PBS (blocking solution) was used for blocking at RT for 1 h, or at 4°C overnight. Primary antibodies, including SOX2 (1: 500, Bio‐techne, AF2018), SOX9 (1: 500, Sigma, AB5535), E‐cadherin (1: 1,500, Thermo Fisher Scientific, 13‐1900) and CD44 (1:100, Thermo Fisher Scientific, 14‐0441‐82), in blocking solution were used at 4°C overnight. After washing off the primaries, the secondary antibodies in 0.1% Triton‐X in PBS were used at 4°C overnight. The following day, DAPI (Sigma, D9542) staining was performed in 0.1% Triton‐X in PBS at RT for 20 min. After three washes in PBS, Fluoromount™ Aqueous Mounting Medium (Sigma, F4680) was used for mounting. Samples were not blinded prior to imaging or analysis. Confocal z stacks or single planes were acquired using Leica SP8 at an optical resolution of 1,024 × 1,024 at 40x. Single plane images are shown. Images were processed using ImageJ (version 2.0.0).

### Lentiviral production

HEK293T cells were grown in 10‐cm dishes to 80% confluency before transfection with the lentiviral vector (10 μg) with packaging vectors including pMD2.G (3 μg, Addgene plasmid # 12259), psPAX2 (6 μg, Addgene plasmid # 12260) and pAdVAntage (3 μg, E1711, Promega) using Lipofectamine 2000 Transfection Reagent (11668019, Thermo Fisher Scientific) according to the manufacturer's protocol. After 16 h, medium was refreshed. Supernatant containing lentivirus was harvested at 24 and 48 h after medium refreshing and pooled together. Supernatant was centrifuged at 300 *g* to remove cell fragments and passed through 0.45 μm filter. Lentivirus containing > 10 kb length insert (inducible CRISPRi) was concentrated using AVANTI J‐30I centrifuge (Beckman Coulter) with JS‐24.38 swing rotor at 72,000 *g* for 2 h at 4°C and pellets were dissolved in 200 μl PBS. Other lentivirus, including individual gRNAs, constructively active β‐catenin and SOX9 inducible overexpression, were concentrated using Lenti‐X™ Concentrator (631232, Takara) and pellets were dissolved in 400 μl PBS.

### gRNA library cloning

gRNA plasmid (Addgene #167936) was linearized with BbsI‐HF restriction enzyme overnight at 37°C. Linearized plasmid was gel purified with QIAquick^®^ Gel Extraction Kit (28704, Qiagen).

The top 5 gRNA sequences for each targeted gene and #0‐#32 non‐targeting control gRNA sequences were selected from (Horlbeck *et al*, 2016). The gRNA library sequences are listed in Dataset EV1. Twenty‐five nucleotide homology sequences flanking the BbsI cleavage sites of the gRNA plasmid were added at 5′ and 3′ ends of the gRNA sequences for cloning. Oligos were purchased as oPools™ Oligo Pools from IDT at 10 pmol per oligo scale.

A 1.0 pmol of oligo pool and 0.005 pmol linearized plasmid were mixed with NEBuilder^®^ HiFi DNA Assembly Master Mix (E2621, New England BioLabs) according to the manufacturer's instructions. The mixture was incubated at 50°C for 1 h and cooled down on ice. Three transformation reactions were performed with 2 μl of the reaction mixture in 50 μl Stellar™ Competent Cells (636763, Takara) each according to the manufacturer's manual. A small fraction of the competent cells was plated to calculate the amount needed for 500X representation per gRNA as seeding in Maxiprep. A total of 10 colonies were randomly picked, minipreped (27104, Qiagen) and sequenced. No repetitive gRNA sequence or backbone sequence were observed. At the same time, the same amount of linearized vector was transformed in the same way for background control (colony number < 5% of the library construction transformation). gRNA library pool plasmid was harvested using EndoFree Plasmid Maxi Kit (12362, Qiagen). gRNA library quality was subsequently checked by next‐generation sequencing (NGS). Primer sequences used are listed in Dataset EV8.

### Lentiviral production for CRISPRi gRNA library

The gRNA library packaging was slightly modified from a previous method reported (Tian *et al*, 2019). Two 15‐cm dishes with 80–90% confluent HEK293T cells were used for transfection. 10 μg CRISPRi gRNA library plasmid, pMD2.G (2.2 μg, Addgene plasmid # 12259), psPAX2 (4 μg, Addgene plasmid # 12260) and pAdVAntage (1.6 μg, E1711, Promega) were diluted into 2 ml Opti‐MEM I Reduced Serum Medium (51985034, Thermo Fisher Scientific). 250 μl Lipofectamine 2000 Transfection Reagent (11668027, Thermo Fisher Scientific) was added into 2 ml Opti‐MEM and incubated at room temperature for 5 min. The diluted DNA mixture was added to the diluted Lipofectamine solution, inverted several times to mix, and incubated at room temperature for 15 min. The transfection mixture was then distributed dropwise to each 15‐cm dish with HEK293T cells and mixed well. The next morning, medium was refreshed with DMEM/F12 with 10% FBS. Two days later, the medium was harvested, spun down and filtered through 0.45 μm filters. Lenti‐X™ Concentrator (631231, Takara) was added to the supernatant at 1: 3 (v/v) ratio. After 48 h, lentivirus was pelleted at 1,500 *g* for 45 min at 4°C. Lentivirus pellets were resuspended with 20 ml of self‐renewing medium with ROCK inhibitor (ROCKi, Y‐27632, 10 μM, 688000, Merck), aliquoted and stored in −80°C before use.

### CRISPRi screen

Lentivirus titration was first performed to determine the amount of CRISPRi gRNA lentivirus needed for the screen. BRC1915 and HDBR‐13393 inducible CRISPRi parental organoid lines were dissociated with TrypLE (Thermo Fisher Scientific, 12605028) at 37°C for 10–20 min (with pipette trituration every 5 min). Organoid single cells were counted and resuspended to achieve 100 K cells/500 μl of self‐renewing medium with ROCKi in each 24‐well plates well. A serial gradient of the CRISPRi gRNA library lentivirus was added and incubated at 37°C overnight. The next morning, organoid cells were collected, washed twice with PBS and seeded back to 2x 24‐well plates well/condition. Flow cytometry analysis was performed after 3 days to determine the amount of virus needed to reach 10–30% of infection rate per 100 K cells per 500 μl of medium.

BRC1915 and HDBR‐13393 inducible CRISPRi parental organoid lines were expanded and dissociated with TrypLE at 37°C for 10–20 min (with pipette trituration every 5 min). 4.2 million and 5 million organoid cells from each parental organoid lines were used for infection respectively. Organoid cells were counted and resuspended at 100 K cells per 500 μl of medium. The infection was performed in a 15‐cm dish with the appropriate amount of lentivirus determined by titration. Organoid cells were collected the next morning, washed and seeded back to Matrigel with 50 K cells/50 μl Matrigel dome in six‐well plates with 8 Matrigel domes per well. Self‐renewing medium with ROCKi was supplemented for 3 days before organoids were dissociated with TrypLE and prepared for cell sorting. The TagRFP^+^EGFP^+^ double positive population was harvested. Organoid cells from BRC1915 line were divided into 3 parts, each with 983x cell representation per gRNA. One part was snap frozen for gRNA abundance analysis and the two remaining parts were separated as technical replicates and seeded back into Matrigel with 10 K cells/50 μl Matrigel dome in 6‐well plates with 8 Matrigel domes per well. Organoid cells from HDBR‐13393 were divided into 2 parts, each with 480x representation per gRNA. One part was snap frozen for gRNA abundance analysis, and the other part was seeded back to Matrigel with 10 K cells/50 μl Matrigel dome in six‐well plates with 8 Matrigel domes per well. Organoid cells were cultured in self‐renewing medium with ROCKi for 8 days before Dox (Doxycycline hyclate, 2 μg/ml, Merck, D9891) and TMP (Trimethoprim, 10 μM, Merck, 92131) were administered and cultured for 2 weeks further. Organoids were passaged (physically split by breaking into pieces) twice during this time. Organoid cells were then dissociated into single cells and prepared for flow cytometry cell sorting. TagRFP^high^EGFP^+^ and TagRFP^low^EGFP^+^ cells were sorted, harvested separately and snap frozen for gRNA abundance analysis.

Genomic DNA was isolated with Macherey‐Nagel™ NucleoSpin™ Blood kit (740951.50, Macherey‐Nagel) according to the manufacturer's instruction. A 2 μg of genomic DNA for BRC1915 organoid line and 1 μg genomic DNA for HDBR‐N 13393 organoid line was used for PCR amplification of gRNA for NGS. NEBNext Ultra II Q5 Master Mix (M0544S, New England BioLabs) and primers (Dataset EV8) were used for amplification with 29 cycles. PCR products were gel purified with Monarch^®^ DNA Gel Extraction Kit (T1020S, New England BioLabs) according to manufacturer's instructions and sent for NGS at the Gurdon Institute NGS core using an Illumina HiSeq 1500.

### CRISPRi screen analysis

Guide abundances were quantified by mapping reads present in sequencing data to the CRISPRi library sequences. The initial nucleotide was omitted from both library sequences and reads, as the initial base of a read is often not certain. Mapped read counts are available in Dataset EV2. MAGeCK (Li *et al*, 2014) was used to determine significantly enriched or depleted genes. As MAGeCK expects the majority of genes targeted to have no effect, the 33 non‐targeting guides were bootstrapped to form 102 synthetic genes by drawing five unique random guides from the non‐targeting group. MAGeCK version 0.5.9.3 was run with “‐‐remove‐zero both” and “‐‐remove‐zero‐threshold 1” options. CRISPRi screen results are summarised in Dataset EV9.

### CRISPRi screen hit validation

The two most effective gRNA sequences in the screen for *MYBL2*, *IRF6*, *ARID5B* and *ZBTB7B* were selected (Dataset EV8). These gRNAs were individually cloned into the gRNA plasmids and packaged into lentivirus as described above.

Three independent inducible CRIPSRi parental organoid lines were transduced individually with specific gRNAs. TagRFP and EGFP double positive organoid cells were sorted and seeded in Matrigel with 2,000–3,000 cells per 50 μl Matrigel (for gRNAs targeting the same gene, the same number of cells were used from each parental organoid line). Organoid cells were cultured with self‐renewing medium plus ROCKi for 8 days before Dox and TMP were administered for 5 days. One‐third of the organoids was harvested for mRNA isolation to check for targeted gene knock‐down. RNA extraction was performed using RNeasy Mini Kit (74104, Qiagen) with RNase‐Free DNase Set (Qiagen) according to the manufacturer's instructions. Reverse transcription was performed using MultiScribe™ Reverse Transcriptase system (4311235, Thermo Fisher Scientific) according to the manufacturer's instructions. qRT–PCR primers for each target genes are listed in Dataset EV8. One‐third of the organoids was used for EdU incorporation assay with 1‐h pulse labelling, fixed and staining with Click‐iT™ EdU Cell Proliferation Kit for Imaging (C10340, Thermo Fisher Scientific) according to the manufacturer's instructions. The rest of the organoids were passaged at 1 to 3 ratio till Passage #5, or the line was lost.

### *SOX2* , *SOX9* and *ETVs* double knock‐down RNA‐Seq

BRC1915 and HDBR‐N 13393 inducible CRISPRi parental organoid lines were used for *SOX2* and *SOX9* knock‐down experiments. BRC1915 and BRC2174 inducible CRISPRi parental organoid lines were used for *ETV4* and *ETV5* double knock‐down. For each parental line, NT gRNAs (#1, #2), *SOX2* gRNAs (#2, #8), *SOX9* gRNAs (#1, #7) and *ETV* gRNAs (#1, #2, both containing gRNAs targeting to *ETV4* and *ETV5* in the same vector) were introduced via lentivirus transduction (gRNA sequences in Dataset EV8). TagRFP and EGFP double positive cells were sorted, replated and expanded. Organoids were treated with Dox and TMP for 5 days, before harvesting for RNA extraction, which was performed using RNeasy Mini Kit (Qiagen) with RNase‐Free DNase Set (Qiagen) according to the manufacturer's instructions.

RNA was quantified by NanoDrop (Thermo Fisher Scientific) and Tape Station (Agilent) with High‐Sensitivity RNA Screen Tape (5067‐5579, Agilent). Half of the RNA was used for cDNA synthesis and qRT–PCR analysis to validate *SOX2*, *SOX9* and *ETVs* knock‐down effect. The other half of the RNA was sent for Eukaryotic RNA‐seq library preparation and sequencing (PE150) at Novogene (Cambridge, UK).

### RNA‐Seq data analysis

Raw sequencing data were aligned to human genome (hg38) using STAR aligner (v 2.7.1a). Sequencing reads were counted using FeatureCount (v 2.0.0). DESeq2 was used for differential gene expression analysis. Differentially expressed (DE) gene lists were generated using DESeq2 packaged with a cut‐off of fold change ≥ 2 and FDR ≤ 0.05 for *SOX2* KD and *SOX9* KD and a cut‐off of fold change ≥ 1.5 and FDR ≤ 0.05 for *ETVs* KD. *SOX2* KD, *SOX9* KD, ETVs KD or NT control with different gRNAs of the same organoid line were first grouped for DE gene analysis. DE genes shared by two independent organoid lines were taken as the most robust DE gene list for downstream analysis. Differentially expressed genes were used for GO analysis with ShinyGO v0.61 (http://bioinformatics.sdstate.edu/go/).

### Comparing SOX9 targets with human foetal lung scRNA‐Seq data

Upregulated and downregulated genes in *SOX9* knock‐down DE gene list and SOX9 direct transcriptional target list were used to score epithelial cells from 9 to11 pcw human foetal lungs (preprint: He *et al*, 2022), using Scanpy's tl.score_genes function.

### SOX9, ETV4 and ETV5 TaDa

Four organoid lines (HDBR‐N 13393, BRC1915, BRC1929 and BRC1938) were used for SOX9, ETV4 and ETV5 TaDa experiments. For each organoid line, 4 × 10^5^ organoid single cells were prepared by the aforementioned method and resuspended in 500 μl self‐renewing medium with ROCKi in one 24‐well plates well. 40 μl of SOX9, ETV4, ETV5 or Dam‐only TaDa lentivirus was added per well and incubated overnight. The next morning, organoid single cells were harvested and washed twice with PBS before being resuspended with 200 μl of Matrigel and seeded back to four 24‐well plates wells. After 48–72 h, organoids were harvested for genomic DNA isolation.

### DamID‐seq

DamID samples were processed as described previously and prepared for Illumina sequencing with an adapted TruSeq protocol (Marshall *et al*, 2016). All sequencing runs were performed as single end 50 bp reads using an Illumina HiSeq 1500, or single end 100 bp reads using an Illumina NovaSeq 6000 from the Gurdon Institute NGS Core facility.

### DamID‐seq data processing

Analysis of the raw fastq files was performed with the damidseq pipeline script (Marshall & Brand, 2015) that maps reads to an indexed bowtie2 genome, bins into GATC‐fragments according to GATC‐sites and normalises reads against a Dam‐only control. For our data, the fastq files were mapped to the GRCh38 genome assembly (hg38). The different patient‐derived organoid samples were treated as individual replicates. For each replicate, the signal from the Dam‐fusion samples was normalised against its own Dam‐only sample with a modified version of the damidseq_pipeline (RPM normalisation, 300 bp bin size). Binding intensities were quantile normalised across all replicates for the same Dam‐fusion and subsequently averaged and inversed (“unlog”). Files were converted to the bigwig file format with bedGraphToBigWig (v4) for visualisation with the Integrative Genomics Viewer (IGV) (v2.4.19).

### Peak calling and analysis

Macs2 (v2.1.2) was used to call broad peaks for every Dam‐fusion and Dam‐only pair using the bam files generated by the damidseq_pipeline. Peaks were selected when present in all replicates for a particular experimental condition with a FDR < 10^−50^. Overlapping genomic features were identified by uploading the peak coordinates to the GREAT online platform (http://great.stanford.edu/public/html/). Motif analysis was performed using HOMER (v 4.11) analysis with findMotifsGenome.pl function. Genomic occupancy feature annotation was performed using HOMER (v 4.11) analysis with annotatePeaks.pl function. Correlation analysis of different TaDa results was performed using deepTools (v 3.5.0) multiBigwigSummary function.

### *In situ* hybridization chain reaction (*in situ* HCR)

*In situ* HCR v3.0 was performed according to the manufacturer's procedure (Molecular Instruments). A set of DNA HCR probes were designed according to the protocol (Choi *et al*, 2018), and the HCR amplifiers with buffers were purchased from Molecular Instruments. Sequence information for the DNA HCR probes for *SOX9*, *ETV4*, *ETV5*, *LGR5* and *NOTUM* mRNA targets are listed in Dataset EV10. In brief, 14 μm frozen human tissue sections were rinsed in nuclease‐free water and prehybridised for 10 min in 37°C humidified chamber. Each DNA HCR probe was diluted to 2 pmol and incubated with the tissues at 37°C overnight. After a series of washes in probe wash buffer, the tissue slices were incubated with 6 pmol of HCR amplifiers at room temperature overnight for amplification reaction. The HCR amplifier comprised of two hairpins, B1 and B2, labelled with Alexa 546 and 647, respectively, were snap‐cooled separately at 3 μM before adding to the tissue sections. After removing excess hairpins in 5X SSCT (sodium chloride sodium citrate) buffer, nuclei were stained with DAPI. Samples were not blinded prior to imaging or analysis. Images were collected on a Leica SP8 confocal microscope.

### Western blot

Organoids were harvested and washed twice with Advanced DMEM/F12 and twice with PBS, before organoids were re‐suspended in 100–200 μl of RIPA buffer with protease inhibitor (1x, Thermo Fisher Scientific, 78440) added. Organoid suspension was incubated for 30 min on ice, with strong vortex every 5 min. Cell pieces and debris were removed by centrifugation at 21,300 *g*. Supernatant was harvested. Protein concentration was measured by BCA assay (Thermo Fisher Scientific). Equal amount of each protein sample was mixed with Sample Buffer (Bio‐rad) and beta‐mercaptoethanol (Merck, M6250) according to the manufacturer's protocol. Mixture was heated at 95°C for 5 min and cooled down to room temperature.

Samples were separated on a 4–12% SDS–PAGE and transferred to nitrocellulose membranes. Proteins were detected by incubation with primary antibodies, SOX9 (1: 1,000, Merck, AB5535) and β‐Actin (1: 5,000, Merck, A3854) at 4°C overnight and subsequently secondary antibodies, donkey anti‐Mouse IRDye 800CW (1: 1,000, AbCam, ab216774) and donkey anti‐Rabbit IRDye 680RD (1: 1,000, AbCam, ab216779). Protein bands were visualised using Li‐Cor Odyssey system.

### Molecular cloning

Inducible CRIPSRi and gRNA plasmids (Addgene: #167935 and #167936, respectively) were previously deposited at Addgene. The TaDa Dam‐only plasmid was generated by In‐fusion cloning of mNeonGreen and *E*.*coli* Dam methylase into a linearized plasmid pHR‐SFFV‐dCas9‐BFP‐KRAB (a gift from Stanley Qi & Jonathan Weissman, Addgene plasmid # 46911), with MluI and SbfI restriction enzymes. SOX9, ETV4 and ETV5 TaDa plasmids (N‐terminal Dam fusion) were generated by In‐fusion cloning of the target gene sequence (amplified from cDNA library) into the linearized TaDa Dam‐only plasmid with NotI restriction enzyme. The constitutively activated β‐catenin overexpression plasmid was generated by In‐fusion cloning of deltaN90 β‐catenin (amplified from cDNA library) and mNeonGreen into the linearized inducible CRISPRi plasmid with XhoI and BamHI restriction enzymes.

## Author contributions

**Dawei Sun:** Conceptualization; data curation; formal analysis; investigation; methodology; writing – original draft; writing – review and editing. **Oriol Llora Batlle:** Software; formal analysis. **Jelle van den Ameele:** Software; formal analysis. **John C Thomas:** Software; formal analysis. **Peng He:** Software; formal analysis. **Kyungtae Lim:** Validation; investigation; methodology. **Walfred Tang:** Software; formal analysis. **Chufan Xu:** Validation; investigation; methodology. **Kerstin B Meyer:** Supervision; funding acquisition; writing – review and editing. **Sarah A Teichmann:** Supervision; funding acquisition. **John C Marioni:** Supervision; funding acquisition. **Stephen P Jackson:** Supervision; funding acquisition; writing – review and editing. **Andrea H Brand:** Supervision; funding acquisition; writing – review and editing. **Emma L Rawlins:** Conceptualization; supervision; funding acquisition; writing – review and editing.

## Disclosure and competing interests statement

Dr Sarah Teichmann is a member of the Scientific Advisory Board for the following companies: Biogen, Foresite Labs, GSK, Qiagen, CRG Barcelona, Jax Labs, SciLife Lab, Allen Institute. She is a consultant for Genentech and Roche. She is co‐founder of Transition Bio and a member of the Board.

## Supporting information



Expanded View Figures PDF

Dataset EV1

Dataset EV2

Dataset EV3

Dataset EV4

Dataset EV5

Dataset EV6

Dataset EV7

Dataset EV8

Dataset EV9

Dataset EV10

Source Data for Expanded View

PDF+

Source Data for Figure 1

Source Data for Figure 2

Source Data for Figure 3

Source Data for Figure 4

## Data Availability

*SOX9* and *SOX2* CRISPRi knock‐down RNA‐seq data, *ETV4*/*ETV5* CRISPRi double knock‐down RNA‐seq data and SOX9/ETV4/ETV5 targeted DamID data have been deposited to GEO (GSE189885; https://www.ncbi.nlm.nih.gov/geo/query/acc.cgi?acc=GSE189885) and are publicly available. Any additional information required to reanalyse the data reported in this paper is available from the corresponding author upon request.
