# Supplementary material for: SOX9 maintains human foetal lung tip progenitor state by enhancing WNT and RTK signalling
Source: EMBO J. 2022 Sep 19;41(21):e111338. doi: 10.15252/embj.2022111338 (PMC9627674; doi:10.15252/embj.2022111338)
Supplement: Supplementary file 1 — Expanded View Figures PDF [file EMBJ-41-e111338-s017.pdf]

Expanded View Figures

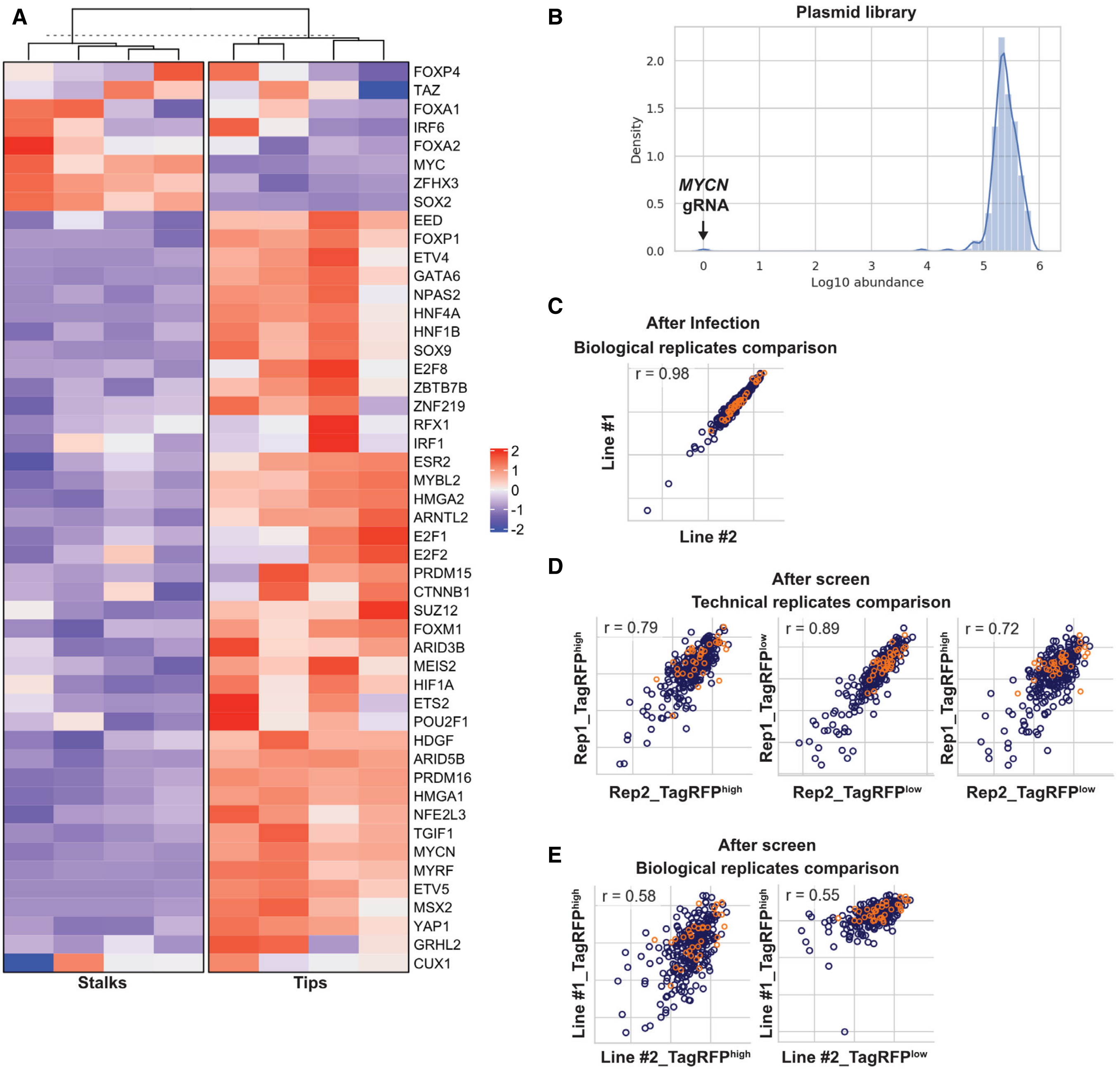

Figure EV1. CRISPRi screen quality control.

A Expression levels heatmap of the selected transcription factors in the developing human foetal lung tip progenitor and stalk cells. Data from Nikolić *et al* (2017).

B gRNA abundance distribution of the CRISPRi library after cloning into the plasmid vector. One gRNA targeting *MYCN* was missing; likely due to a gRNA synthesis issue.

C Pearson correlation of gRNA abundance between different samples indicated in axes. Between two independent CRISPRi parental lines 3 day after lentiviral transduction. $R = 0.98$ indicated great consistency of lentiviral transduction.

D Pearson correlation of gRNA abundance between technical replicates (Rep1 and Rep2). Great consistency was observed between TagRFP^{high} and TagRFP^{low} populations.

E Pearson correlation of gRNA abundance between biological replicates. A lower correlation was observed reflecting the variation of human tissue samples.

Data information: Orange circles in (C–E) represent non-targeting control gRNAs.

 © 2022 The Authors

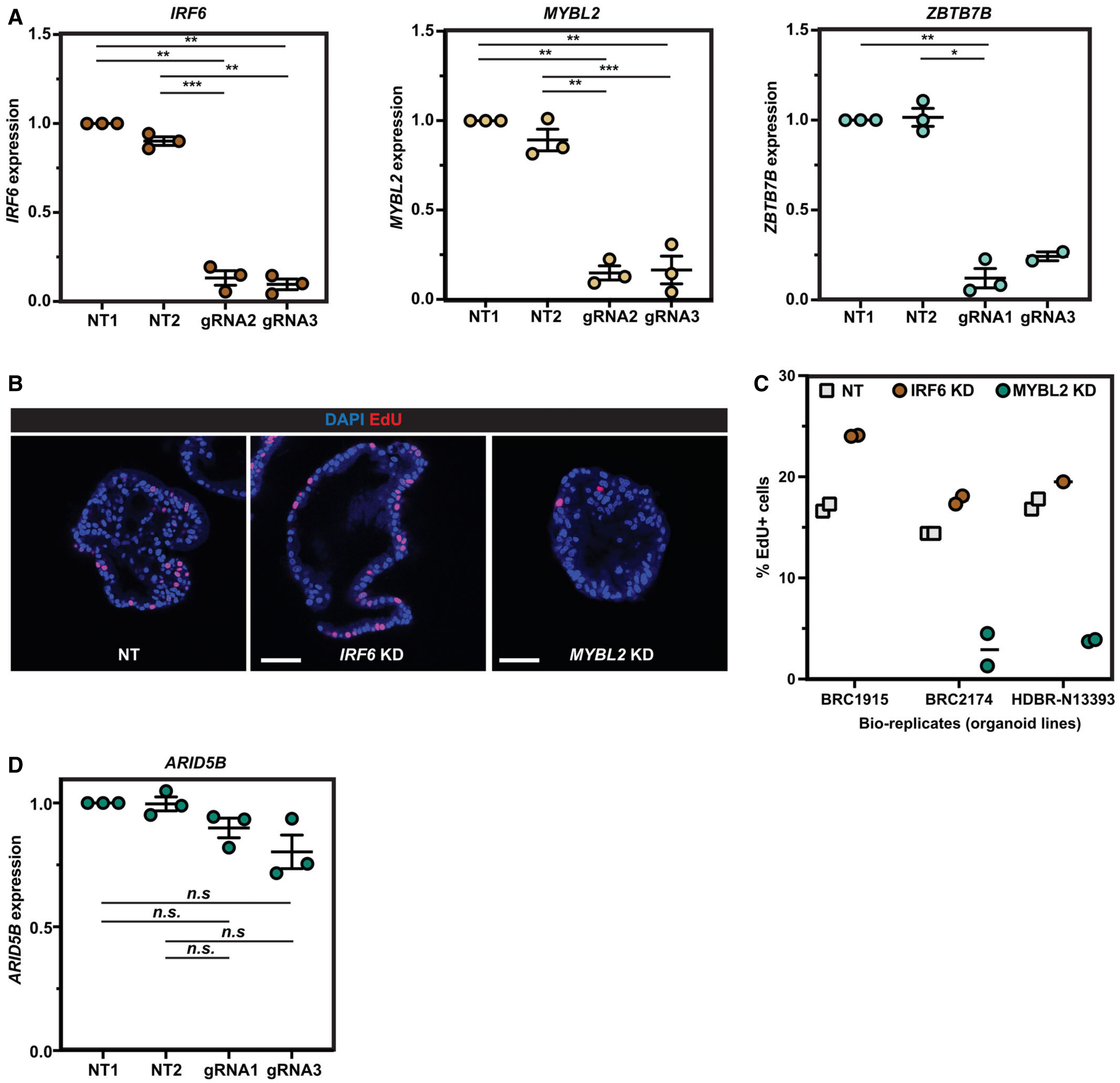

Figure EV2. Validation of the CRISPRi screen results.

A qRT–PCR results showing the targeted genes (*IRF6*, *MYBL2* and *ZBTB7B*) were efficiently knocked down by the inducible CRISPRi system using the gRNAs selected from the CRISPRi gRNA library.

B Representative EdU staining images of non-targeting gRNA control and *IRF6* or *MYBL2* knock-down experiments.

C Quantification of the percentage of EdU^+ cells in each of three parental organoid lines used with non-targeting control, *IRF6* knock-down and *MYBL2* knock-down. n = 1,649, 1,705, 3,548 cells were scored for NT controls. n = 2,517, 950, and 1,313 cells were scored for *IRF6* gRNAs. n = 1,098 and 1,306 cells were scored for *MYBL2* gRNAs.

D qRT–PCR results showing *ARID5B* was not knocked down by the inducible CRISPRi system using the gRNAs selected from the CRISPRi gRNA library.

Data information: Error bars: mean ± SEM. Statistical analysis was using the two-tailed paired t-test. P-values are reported as follows: $*P < 0.05$, $**P < 0.01$, $***P < 0.001$ and n.s. non-significant. N = 3 organoid lines (biological replicates) used for each panel.
Source data are available online for this figure.

© 2022 The Authors

Figure EV3. *SOX2* and *SOX9* knock-down resulted in different transcriptome changes.

A Unsupervised hierarchical clustering of non-targeting control, *SOX2* knock-down and *SOX9* knock-down RNA-Seq results.

B Venn diagram showing minimal overlap of differentially expressed genes after *SOX2* knock-down in two different parental organoid lines. Overlapping DE genes were labelled in boxes.

C qPCR of selected DE genes from *SOX9* RNA-seq data following *SOX9* knock-down in a further 2 independent organoid lines. Cells harvested 5 days after knock-down. Error bars: mean ± SEM. Statistical analysis was using the two-tailed paired *t*-test. *P*-values are reported as follows: $^{**}P < 0.01$, $^{***}P < 0.001$. $N = 3$ bio-replicates (Organoid line BRC2174 with two different NT gRNAs and two different SOX9 gRNAs, and Organoid line BRC2136 with 1 NT gRNA and 1 SOX9 gRNA) were used.

D Sashimi plot to visualise splicing junction of NT control and *SOX9* KD. Upper panel: Sashimi plot was used to visualise splicing junction information in non-targeting gRNA control and *SOX9* knock-down groups. Junctional reads between intron #1 and exon #2 were only observed in *SOX9* knock-down groups and not in non-targeting gRNA control groups. Lower panel: major SOX9 domains in relation to the *SOX9* genomic locus. Exon #1 contains DIM and part of the HMG domain. DIM, dimerization domain. HMG, high-mobility group domain. PQA, proline-glutamine-alanine repeats domain. TA, transactivation domain.

E Heatmap of gene expression from representative GO terms: cell division and small molecule metabolism together with gene expression of upregulated non-lung lineage genes. OL, organoid line.

F Selected GO enrichment in DE genes after *SOX9* knock-down.

Source data are available online for this figure.

 © 2022 The Authors

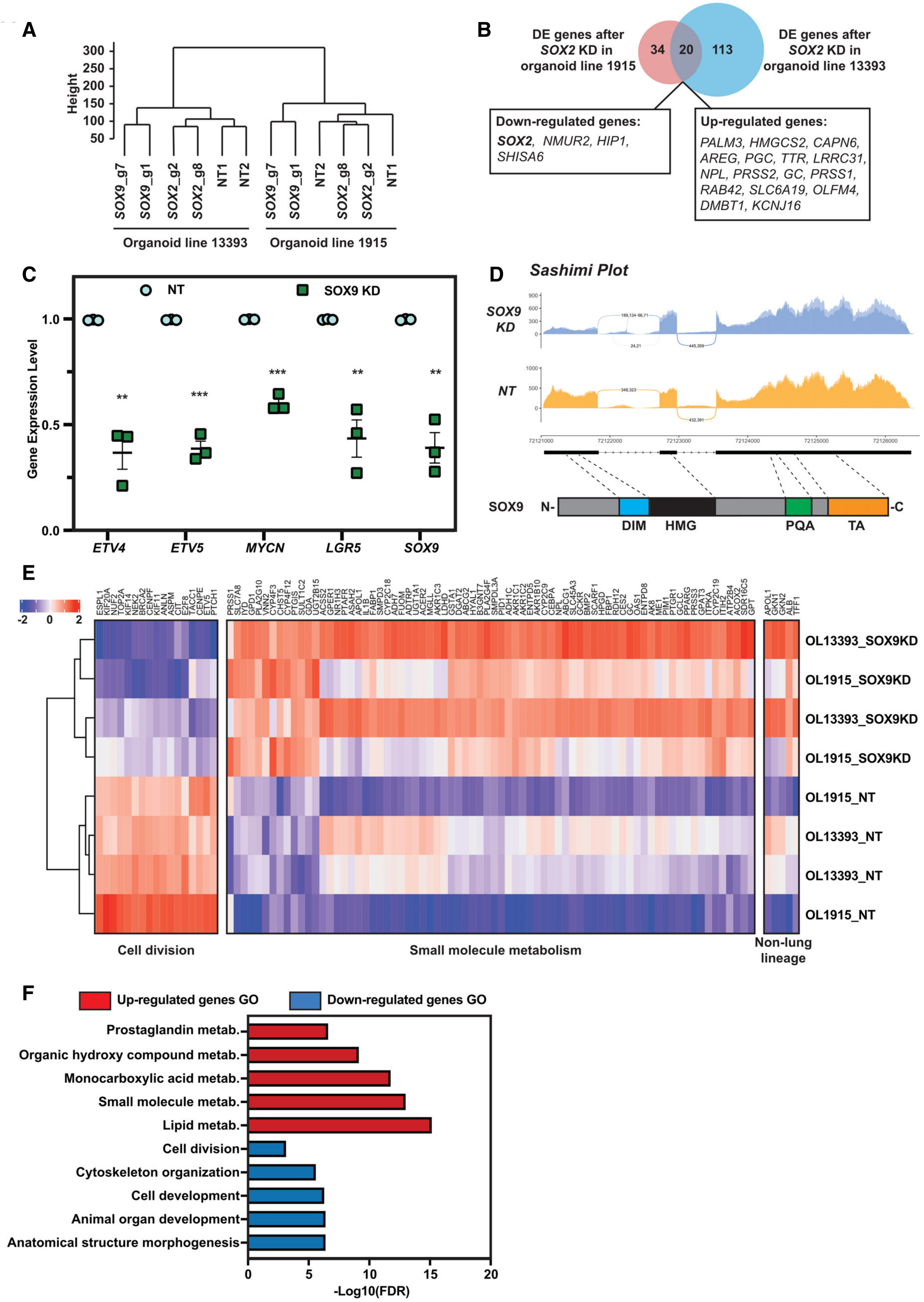
A
Height
300
250
200
150
100
50
SOX9_g7
SOX9_g1
SOX2_g2
SOX2_g8
NT1
NT2
SOX9_g7
SOX9_g1
NT2
SOX2_g8
SOX2_g2
NT1
Organoid line 13393
Organoid line 1915
B
DE genes after SOX2 KD in organoid line 1915
34
20
113
DE genes after SOX2 KD in organoid line 13393
Down-regulated genes:
SOX2, NMUR2, HIP1, SHISA6
Up-regulated genes:
PALM3, HMGCS2, CAPN6, AREG, PGC, TTR, LRRC31, NPL, PRSS2, GC, PRSS1, RAB42, SLC6A19, OLFM4, DMBT1, KCNJ16
C
NT
SOX9 KD
Gene Expression Level
1.0
0.5
0.0
**
***
***
**
**
ETV4
ETV5
MYCN
LGR5
SOX9
D
Sashimi Plot
SOX9 KD
NT
SOX9
N-
-C
DIM
HMG
PQA
TA
E
-2
0
2
OL13393_SOX9KD
OL1915_SOX9KD
OL13393_SOX9KD
OL1915_SOX9KD
OL1915_NT
OL13393_NT
OL13393_NT
OL1915_NT
Cell division
Small molecule metabolism
Non-lung lineage
F
Up-regulated genes GO
Down-regulated genes GO
Prostaglandin metab.
Organic hydroxy compound metab.
Monocarboxylic acid metab.
Small molecule metab.
Lipid metab.
Cell division
Cytoskeleton organization
Cell development
Animal organ development
Anatomical structure morphogenesis
0
5
10
15
20
-Log10(FDR)

Figure EV3.

Figure EV4. SOX9 directly activates tip cell genes and represses secretory cell genes.

A Summary of enriched TF binding motifs in SOX9 TaDa peaks. The SOX motif was enriched, indicating the SOX9 TaDa faithfully identified SOX9 binding sites across the genome.

B SOX9 direct transcriptional target enrichment in human foetal lung scRNA-seq data. All SOX9 direct transcriptional targets were used for scoring. Similar to Fig 2H, SOX9 directly activated targets were enriched in tip progenitor cells (left panel), whereas SOX9 directly repressed targets were enriched in secretory cell lineages (right panel).

C *SHH* was co-expressed with *SOX9* in human foetal lung tip progenitor cells. *SOX9* in yellow and *SHH* in red. No-probe controls are shown in the right panel.

D *LEF1* and *WIF1* were co-expressed with *SOX9* in human foetal lung tip progenitor cells. *SOX9* in red and *LEF1* (left panel) and *WIF1* (right panel) in yellow.

E Lentiviral construct design for overexpressing SOX9 in human foetal lung progenitor cells.

F Representative images showing organoid morphology does not change after 3 days of SOX9 overexpression. SOX9 overexpressed organoid indicated with arrow.

G qRT–PCR results showing that 3 days of SOX9 overexpression led to *ETV5* and *MYCN* transcription being significantly upregulated, however, *ETV4* and *CFTR* were not changed. $N = 4$ organoid lines (bio-replicates) were used. Error bars: mean ± SEM. Two-tailed Student's *t*-tests were performed. *P*-values are reported as follows: $*P < 0.05$; $***P < 0.001$.

Data information: Scale bars denote 50 μm (C, D) and 100 μm (F).
Source data are available online for this figure.

 © 2022 The Authors

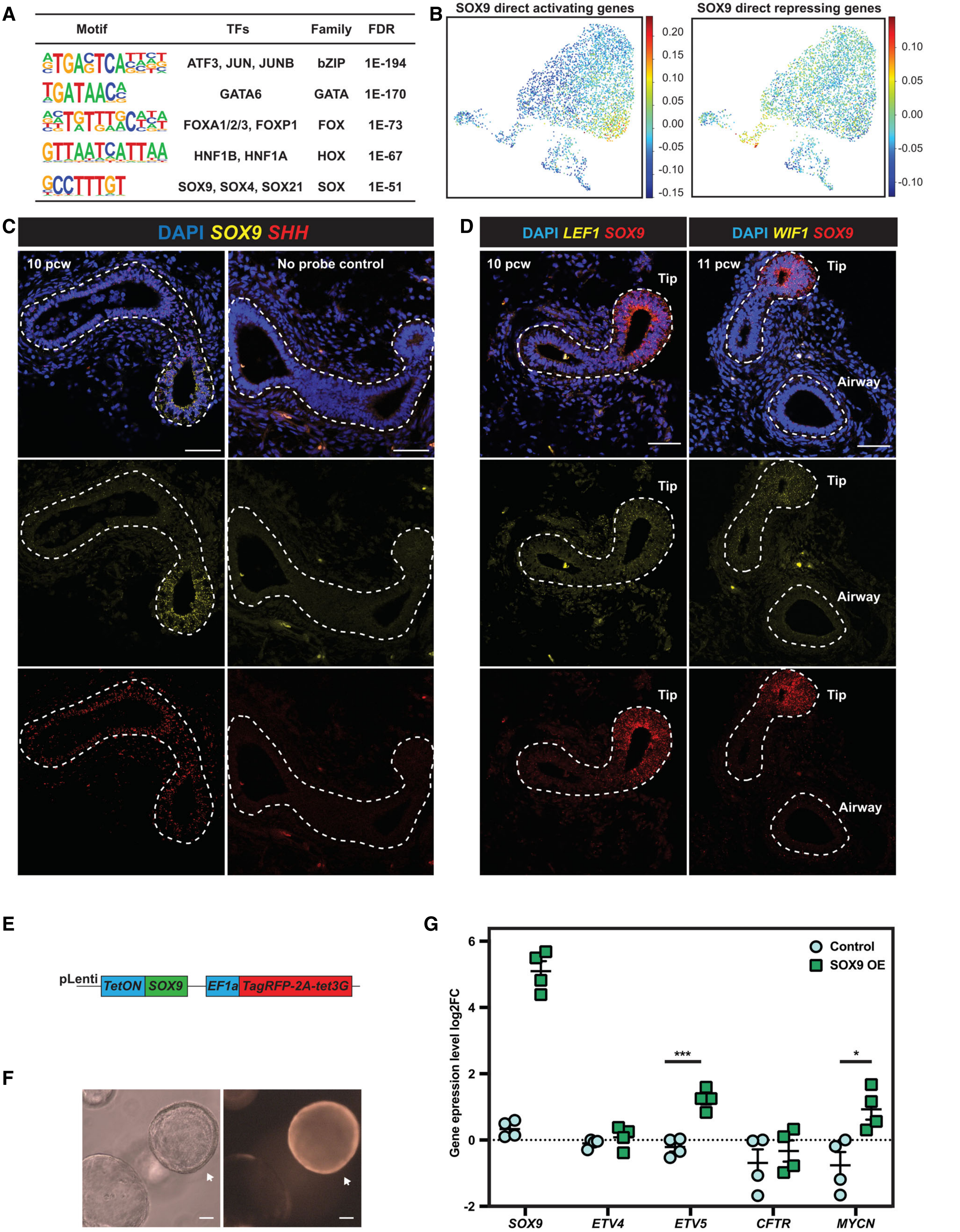
A
Motif
TFs
Family
FDR
ATF3, JUN, JUNB
bZIP
1E-194
GATA6
GATA
1E-170
FOXA1/2/3, FOXP1
FOX
1E-73
HNF1B, HNF1A
HOX
1E-67
SOX9, SOX4, SOX21
SOX
1E-51
B
SOX9 direct activating genes
SOX9 direct repressing genes
C
DAPI SOX9 SHH
10 pcw
No probe control
D
DAPI LEF1 SOX9
DAPI WIF1 SOX9
10 pcw
11 pcw
Tip
Airway
E
pLenti
TetON
SOX9
EF1a
TagRFP-2A-tet3G
F
G
Control
SOX9 OE
Gene epression level log2FC
SOX9
ETV4
ETV5
CFTR
MYCN

Figure EV4.

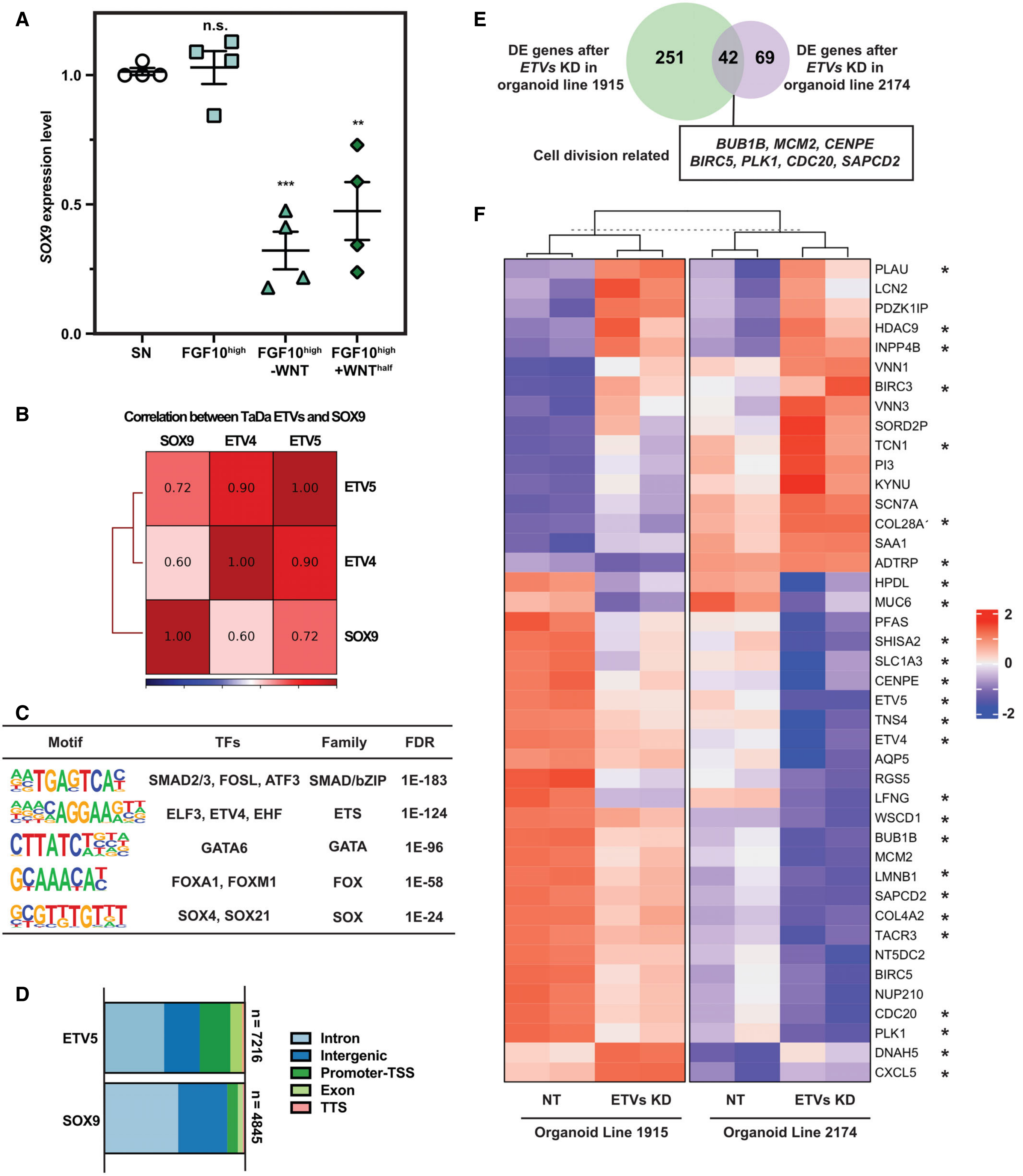

Figure EV5.

 © 2022 The Authors

Figure EV5. Identification of direct ETV5 binding targets.

- A qRT–PCR showing *SOX9* transcription 6 day after FGF10 supplementation (500 ng/ml), or FGF10 supplementation and removal of WNT activators. $N = 4$ different organoid lines. Error bars: mean ± SEM. Statistical analysis was using the two-tailed paired *t*-test. *P*-values are reported as follows: $^{**}P < 0.01$; $^{***}P < 0.001$; n.s. nonsignificant.
- B Pearson correlation of SOX9, ETV4 and ETV5 TaDa. ETV4 and ETV5 TaDa exhibited great consistency.
- C Motifs enriched in ETV5 TaDa peaks. The ETS binding motif was highly enriched.
- D Genomic occupancy annotated features for SOX9 and ETV5 peaks.
- E Venn diagram showing overlap of differentially expressed genes after *ETV4* and *ETV5* double knock-down in two different parental organoid lines. Overlapping DE genes related to cell division by GO analysis are labelled in the box.
- F Heatmap showing expression level of all 42 DE genes after *ETV4*; *ETV5* double knock-down across different organoid lines. Directly regulated genes are marked by asterisks.

Source data are available online for this figure.

© 2022 The Authors
