# Supplementary material for: SOX9 maintains human foetal lung tip progenitor state by enhancing WNT and RTK signalling
Source: EMBO J. 2022 Sep 19;41(21):e111338. doi: 10.15252/embj.2022111338 (PMC9627674; doi:10.15252/embj.2022111338)
Supplement: Supplementary file 18 [file EMBJ-41-e111338-s002.pdf]

THE EMBO JOURNAL

SOX9 maintains human fetal lung tip progenitors by enhancing WNT and RTK signalling

Dawei Sun, Oriol Batlle, Jelle van den Ameele, John Thomas, Peng He, Kyungtae Lim, Walfred Tang, Chufan Xu, Kerstin Meyer, Sarah Teichmann, John Marioni, Stephen Jackson, Andrea Brand, and Emma Rawlins
DOI: 10.15252/embj.2022111338

Corresponding author: Emma Rawlins (elr21@cam.ac.uk)

Review Timeline:

Submission Date:	3rd Apr 22
Editorial Decision:	3rd May 22
Revision Received:	5th Jul 22
Editorial Decision:	28th Jul 22
Revision Received:	11th Aug 22
Accepted:	16th Aug 22

Editor: Ieva Gailite

Transaction Report:

(Note: With the exception of the correction of typographical or spelling errors that could be a source of ambiguity, letters and reports are not edited. Depending on transfer agreements, referee reports obtained elsewhere may or may not be included in this compilation. Referee reports are anonymous unless the Referee chooses to sign their reports.)

Thank you for submitting your manuscript for consideration by The EMBO Journal. We have now received a full set of referee reports on your manuscript, which are included below for your information.

As you will see from the comments, all reviewers appreciate the study, while also indicating a number of concerns that would have to be addressed and clarified before they can support publication of the manuscript. From my side, I find these points reasonable and, based on these positive assessments, I would like to invite you to address the issues raised by the reviewers in a revised manuscript.

We generally allow three months as standard revision time. As a matter of policy, competing manuscripts published during this period will not negatively impact on our assessment of the conceptual advance presented by your study. However, please contact me as soon as possible upon publication of any related work to discuss the appropriate course of action. Should you foresee a problem in meeting this three-month deadline, please let us know in advance and we may be able to grant an extension.

When preparing your letter of response to the referees' comments, please bear in mind that this will form part of the Review Process File and will therefore be available online to the community. For more details on our Transparent Editorial Process, please visit our website: https://www.embopress.org/page/journal/14602075/authorguide#transparentprocess. Please also see the attached instructions for further guidelines on preparation of the revised manuscript.

Please feel free to contact me if you have any further questions regarding the revision. I would be happy to discuss the revision in more detail via email or phone/videoconferencing.

Thank you for the opportunity to consider your work for publication. I look forward to receiving the revised manuscript.

Referee #1:

Here Sun et al, used human lung bud tip organoid models to study transcription factors that control progenitor self-renewal and differentiation programs. The authors used inducible CRISPRi to perform a semi screen to identify transcription factors that control progenitor replication. This screen identified some known regulators of tip progenitor proliferation including SOX9. The authors went on to identify the genomic loci directly bound by SOX9. For this, the authors used DamID based SOX9 genomic

occupancy profiling. They have found numerous genes that are directly bound by SOX9 and also down regulated upon SOX9 knock down. Some of them include regulators of Wnt signaling and other transcription factor such as ETV5. Then they went on to functionally test the role of Wnt signaling and RTK pathways in regulation of SOX9 expression. The authors state that Wnt but not RTK signaling controls SOX9 expression in lung bud tip progenitors. Additionally, the authors tested the interaction between ETV transcription factors and found that SOX9 and ETV5 cooperate to control some target genes in distal tip progenitors.

Overall, this manuscript utilized state of the art tools to study human lung development and the role of transcriptional factors in lung bud tip progenitor self-renewal. Much of the findings from this study have been previously described in mouse lung development and elsewhere. Specifically, the regulation of SOX9 by Wnt and vice versa has been previously shown in multiple tissues. Similarly, the interaction between SOX9 and ETV5 has been described previously. However, to the authors credit, this work provides a technical roadmap for using genetic screens to study human lung development. Therefore, I suggest this manuscript is more suitable for a technical report. Here are some major and minor comments to improve this manuscript.

1. In Fig2G, the authors show DE genes after SOX9 knock-down. It appears that the two organoid lines show significant differences in DE genes. Therefore, it is important to test this on additional organoid lines.

2. There are some GI track genes in Sox9 KD cells. Previous studies have shown that high Wnt signaling in developing lungs activates GI program. Does that mean SOX9 has Wnt dependent and independent functions in lung bud tips?

3. In Fig.3F, the authors show NOTUM expression. Its unclear why the NOTUM came out of the blue? Similarly, the authors show LGR5 expression by FISH. However, it is not clear whether they are important for lung bud tip progenitor replication. Additionally, in Fig. 4I schematic the authors indicate that LGR5 mediating between Wnt and SOX9. Current manuscript does not provide data to support this. Authors could knock down LGR5 and test how that impacts Wnt and SOX9 expression/signaling in distal tip progenitor organoids.

Minor comments:

1. In line 175, test states Fig. 1F-I. There is no panel I.

2.The organoid line numbering is somewhat confusing to the readers. I suggest write - "Organoid line 1915 etc. in Figures.

3. In line 294, change "...human tips..." to "...human lung bud tips...".

Referee #2:

This manuscript reports the use of CRISPR interference (CRISPRi) screening and Targeted DamID (TaDa) in human fetal lung organoids. Although a significant proportion of the paper is methodological, the authors successfully employ current developmental biology knowledge to validate their experimental model and, along the way, they stumbled upon very interesting findings such as the observation that ETV4 and ETV5, two downstream targets for FGF signaling in the embryonic mouse lung, are direct binding targets for SOX9, and that SOX9 and ETVs cooperate to control the self-renewal of tip progenitor cells. Even when the authors were faced with counterintuitive findings such as their observation that SOX9 was not downregulated in the RNA-seq data following CRISPR-mediated SOX9 knockdown, they investigated further and uncovered an alternative transcriptional start site that is unlikely to produce a functional SOX9 protein, thus appropriately explaining their observations. Overall, the approach that the authors report here is, to my knowledge, very novel and paves the way for future exploitation of this exciting model to ask questions that are not only relevant to cell and developmental biology but also to respiratory diseases. The paper is well written, the methods are sufficiently explained, and the data are well analyzed and presented. There are a few minor issues that should be addressed:

1. The in situ hybridization in Fig. S4C shows weak signals. Control probes are also missing
2. Although LGR5 is expressed in human lung adenocarcinoma and has been shown to be expressed in human fetal bud tip progenitors (Hein et al., bioRxiv, 2021), its expression has been mainly shown in mesenchymal cells in the adult mouse lung (Lee et al., Cell, 2017). The authors are encouraged to comment on the difference in the expression pattern between mouse and human.
3. The Discussion needs to be further developed, particularly regarding future directions and the potential use of this model/approach in a clinical context such as drug screening etc. It should also address the limitations of the methodology.
4. Some sentences in the Introduction are short and look truncated

Referee #3:

In this manuscript Sun et al. performed a pooled CRISPRi drop-out screening in primary human lung epithelial tip organoids to

probe the function of 49 transcription factors known to be expressed in these cells. gRNAs targeting several transcription factors were strongly or moderately depleted, including CTNNB1, MYBL2 and SOX9 consistent with their important role in distant tip progenitor cell maintenance/renewal. Interestingly a gRNA targeting IRF6 was enriched suggesting it normally represses self-renewal. SOX9 knockdown led to DE of about 455 genes. As expected SOX9 suppresses premature differentiation of distal epithelial progenitors into airway epithelial cell types but also differentiation into other foregut lineages. SOX9 also suppresses metabolic processes and promotes cell division. SOX9 targeted DamId was used to identify direct SOX9 target genes. 171 direct SOX9 target genes were identified. Interestingly SOX9 regulates Etv4/5 but also several Wnt pathway genes. SOX9 was found to be a direct Wnt target gene but also further enhanced Wnt signaling in a feedforward loop. Etv4/5 double knockdown like SOX9 knockdown negatively affected progenitor renewal. Removal of RTK ligands (EGF, FGF7 and FGF10) did not seem to affect SOX9 expression at least in the presence of other Wnt activators in the culture medium. TaDa analysis of Etv4/5 suggests that Etv4/5 and SOX9 may coregulate certain target genes though through binding at different loci.

Major concerns.
A) In the mouse Fgf10 has been shown to induce Sox9 expression and inhibition of Fgf10 signaling also suppresses Sox9 expression. It is thought that Fgf10 can does this via PI3K AKT signaling to inhibit GSK3β. Could it be that R-spondin-1 and CHIR99021 levels in the culture medium are so high that they compensate for RTK ligand loss? Also 100ng/ml of Fgf10 is usually not sufficient to induce proper Fgf10 signaling.
To test this the authors should try
1) Increase FGF10 to 500ng/ml and probe for Sox9 expression.
2) Increase FGF10 to 500ng/ml and remove or reduce R-spondin-1 and/or CHIR99021 (especially the GSK3β inhibitor CHIR99021).
B) The authors perform a pretty good analysis of which genes are directly and indirectly regulated by SOX9 but fail to do so for Etv4/5. Which genes are differentially expressed in the Etv4/5 double knockdown and what are the direct Etv4/5 target genes and which genes are likely co-regulated by Etv4/5 and Sox9?

Minor concerns
C) Which genes are differentially expressed in the IRF6 knockdown and what are the direct IRF6 target genes?

Referee #1:

Here Sun et al, used human lung bud tip organoid models to study transcription factors that control progenitor self-renewal and differentiation programs. The authors used inducible CRISPRi to perform a semi screen to identify transcription factors that control progenitor replication. This screen identified some known regulators of tip progenitor proliferation including SOX9. The authors went on to identify the genomic loci directly bound by SOX9. For this, the authors used DamID based SOX9 genomic occupancy profiling. They have found numerous genes that are directly bound by SOX9 and also down regulated upon SOX9 knock down. Some of them include regulators of Wnt signaling and other transcription factor such as ETV5. Then they went on to functionally test the role of Wnt signaling and RTK pathways in regulation of SOX9 expression. The authors state that Wnt but not RTK signaling controls SOX9 expression in lung bud tip progenitors. Additionally, the authors tested the interaction between ETV transcription factors and found that SOX9 and ETV5 cooperate to control some target genes in distal tip progenitors.

Overall, this manuscript utilized state of the art tools to study human lung development and the role of transcriptional factors in lung bud tip progenitor self-renewal. Much of the findings from this study have been previously described in mouse lung development and elsewhere. Specifically, the regulation of SOX9 by Wnt and vice versa has been previously shown in multiple tissues. Similarly, the interaction between SOX9 and ETV5 has been described previously. However, to the authors credit, this work provides a technical roadmap for using genetic screens to study human lung development. Therefore, I suggest this manuscript is more suitable for a technical report. Here are some major and minor comments to improve this manuscript.

1. In Fig2G, the authors show DE genes after SOX9 knock-down. It appears that the two organoid lines show significant differences in DE genes. Therefore, it is important to test this on additional organoid lines.

Thank you for this suggestion. To confirm that the gene expression changes that we have been focusing on following *SOX9* knock-down are robust, we used our *SOX9* gRNAs to knock-down *SOX9* in a further two independent organoid lines followed by qPCR for *SOX9*, *ETV4*, *ETV5*, *MYCN* and *LGR5*. As expected, all were significantly depleted following *SOX9* knock-down (Fig. EV3C).

The baseline variation in overall levels of transcription between human organoid lines/stem cells from different donors is rarely discussed. In our RNAseq experiments, we have performed controls for biological variation (2 organoid lines) and technical variation (lentiviral transduction and gRNA variables). This is a robust approach to performing genome-wide assays on human organoid lines that will be of interest to the community. We now discuss this in the discussion section.

2. There are some GI track genes in Sox9 KD cells. Previous studies have shown that high Wnt signaling in developing lungs activates GI program. Does that mean SOX9 has Wnt dependent and independent functions in lung bud tips?

Our data show clearly that SOX9 is a β-catenin target in the developing human lung (Fig. 3G-I). However, SOX9 itself has many direct targets in various signalling pathways including cytokine, FGF, HH, and WNT pathways, strongly suggesting that downstream of SOX9 there are both WNT-dependent and independent functions. Unfortunately, at the moment it is unclear whether the depression of GI genes that we observe following *SOX9* knock-down is WNT-dependent or independent. However, at least two of the GI-related genes are direct SOX9 targets (*APOL1, TFF1*).

The developing lung and intestine have a close relationship and there are several experimental perturbations which result in the expression of intestinal-specific genes in the lung epithelium. Overexpression of a highly active β-catenin-Lef1 fusion protein in the developing mouse lung resulted in transdetermination of lung epithelial lineages to gut, suggesting that β-catenin can promote GI fate (Okubo et al., 2004; DOI: 10.1186/jbiol3). By contrast, loss of β-catenin in the developing mouse lung resulted in loss of Sox9, decreased Nkx2.1, ectopic Sox2 and derepression of GI genes, suggesting that β-catenin is required to repress GI lineage gene expression (Ostrin et al., 2018; DOI: 10.1242/dev.160788).

These somewhat conflicting results mean that the relationship between Wnt signalling and depression of GI genes in the lung is not clear and it is quite difficult to speculate exactly how Sox9 fits in. However, at least two of the GI-related genes are direct SOX9 targets (*APOL1, TFF1*). One parsimonious model, mostly consistent with our data, is that loss of SOX9 causes direct derepression of some GI genes and moreover, results in loss of *LGR5* therefore lowering WNT activity and causing further derepression of GI genes, consistent with Ostrin et al (Ostrin et al., 2018; DOI: 10.1242/dev.160788). However, our organoids are grown in the presence of the strong WNT activator CHIR99021 which acts downstream of the receptor, and it seems unlikely that signalling events at the cell surface would strongly affect the level of WNT activity in cells grown in these conditions. We have now included a short section in the discussion about these interesting possibilities raised by the reviewer.

3. In Fig.3F, the authors show NOTUM expression. Its unclear why the NOTUM came out of the blue? Similarly, the authors show LGR5 expression by FISH. However, it is not clear whether they are important for lung bud tip progenitor replication. Additionally, in Fig. 4I schematic the authors indicate that LGR5 mediating between Wnt and SOX9. Current manuscript does not provide data to support this. Authors could knock down LGR5 and test how that impacts Wnt and SOX9 expression/signaling in distal tip progenitor organoids.

There are several interesting, related, points here:

3.1 – why look at *NOTUM*?

Following our observation that *LGR5* is a direct SOX9 target, we wished to confirm that the human tip cells are indeed experiencing high WNT signalling at the stages of development we are assessing. We therefore stained for *NOTUM* as a known WNT target (Fig. 3F). We have now looked more widely at WNT targets and also included *LEF1* and *WIF1* (Fig. EV4D), confirming that the bud tip cells are Wnt-responsive. Moreover, recent work from the

Spence lab also confirms that human bud tip cells are responding to WNT (Hein et al., 2022; DOI: 10.1016/j.devcel.2022.05.010).

3.2 – is WNT signalling important for bud tip progenitor replication?
We and others have previously shown that WNT activity is required for bud tip progenitor replication (Nikolic et al 2017, doi: 10.7554/eLife.26575; Miller et al., 2018 DOI: 10.1016/j.stemcr.2017.11.012). This is now made clear in the text.

3.3 – can the authors test their model in 4I that LGR5 is mediating between WNT and SOX9 by knocking-down *LGR5*?
This would be an extremely good experiment. Unfortunately, we cannot perform it in our organoid system due to the presence of both RSPO1 and CHIR99021 in our self-renewing organoid growth medium. We have previously shown that both of these factors are required for long-term organoid self-renewal (Nikolic et al 2017, doi: 10.7554/eLife.26575). CHIR99021 activates the WNT pathway downstream of the receptor by inhibiting GSK3B to stabilise B-catenin. Knock-down of *LGR5* in the presence of CHIR99021 would not allow us to test the role of LGR5 in organoid self-renewal.

We could have attempted the *LGR5* knock-down by growing the organoids in RSPO/WNT in place of CHIR99021. However, the Spence lab have recently published a manuscript which shows that *LGR5* is required for efficient bud tip self-renewal using an LGR5 protein blocker in human fetal lung slice cultures and also shRNA knock-down in human lung bud tip organoids (Hein et al., 2022; DOI: 10.1016/j.devcel.2022.05.010). This paper showed clearly that WNT-RSPO2-LGR5 are required for bud tip self-renewal, but it did not link *LGR5* expression to SOX9.

The Hein et al., manuscript is highly complementary to our current study and although we previously referred to it in the discussion, we now also cite it more prominently in the WNT signalling section of the main text.

Minor comments:

1. In line 175, test states Fig. 1F-I. There is no panel I.
Thank you – now corrected.

2.The organoid line numbering is somewhat confusing to the readers. I suggest write - "Organoid line 1915 etc. in Figures.
This has been changed for clarity.

3. In line 294, change "...human tips..." to "...human lung bud tips...".
Corrected – thank you.

Referee #2:

This manuscript reports the use of CRISPR interference (CRISPRi) screening and Targeted DamID (TaDa) in human fetal lung organoids. Although a significant proportion of the paper is methodological, the authors successfully employ current developmental biology knowledge

to validate their experimental model and, along the way, they stumbled upon very interesting findings such as the observation that ETV4 and ETV5, two downstream targets for FGF signaling in the embryonic mouse lung, are direct binding targets for SOX9, and that SOX9 and ETVs cooperate to control the self-renewal of tip progenitor cells. Even when the authors were faced with counterintuitive findings such as their observation that SOX9 was not downregulated in the RNA-seq data following CRISPR-mediated SOX9 knockdown, they investigated further and uncovered an alternative transcriptional start site that is unlikely to produce a functional SOX9 protein, thus appropriately explaining their observations. Overall, the approach that the authors report here is, to my knowledge, very novel and paves the way for future exploitation of this exciting model to ask questions that are not only relevant to cell and developmental biology but also to respiratory diseases. The paper is well written, the methods are sufficiently explained, and the data are well analyzed and presented. There are a few minor issues that should be addressed:

1. The in situ hybridization in Fig. S4C shows weak signals. Control probes are also missing

We have repeated this staining and it is now shown alongside the no-probe control in Figure EV4C. We confirm that *SHH* is weakly expressed in the human embryonic lungs in agreement with another recent publication (Belgacemi et al., 2022; DOI: 10.3390/ijms23095265).

2. Although LGR5 is expressed in human lung adenocarcinoma and has been shown to be expressed in human fetal bud tip progenitors (Hein et al., bioRxiv, 2021), its expression has been mainly shown in mesenchymal cells in the adult mouse lung (Lee et al., Cell, 2017). The authors are encouraged to comment on the difference in the expression pattern between mouse and human.

LGR5 is considered to be a WNT target gene in multiple tissues {Barker:2010cp}. Our manuscript and Hein et al., show that *LGR5* is expressed in human lung epithelial bud tip progenitors (Hein et al., 2022; DOI: 10.1016/j.devcel.2022.05.010). However, *Lgr5* has also been detected in mouse lung bud tip progenitors using RNA profiling methods (Ostrin et al., 2018; DOI: 10.1242/dev.160788). Moreover, in the adult lung tissue an integrated human lung single cell atlas has a low level of *LGR5* expression in fibroblasts and of *LGR6* in smooth muscle (Sikkema et al., 2022; doi.org/10.1101/2022.03.10.483747). This is in agreement with the mouse data in Lee et al., as mentioned by the reviewer. Our interpretation therefore is that the site of LGR5 expression is more likely to vary with developmental stage than with species.

3. The Discussion needs to be further developed, particularly regarding future directions and the potential use of this model/approach in a clinical context such as drug screening etc. It should also address the limitations of the methodology.

We have now included more discussion about the potential use of this approach for respiratory research. We also discuss the limitations of the CRISPR screening approach in general and of performing screens and NGS in primary cell-derived organoid systems with inherent variability.

4. Some sentences in the Introduction are short and look truncated

We have rewritten the introduction with a focus on improving the language usage.

Referee #3:

In this manuscript Sun et al. performed a pooled CRISPRi drop-out screening in primary human lung epithelial tip organoids to probe the function of 49 transcription factors known to be expressed in these cells. gRNAs targeting several transcription factors were strongly or moderately depleted, including CTNNB1, MYBL2 and SOX9 consistent with their important role in distant tip progenitor cell maintenance/renewal. Interestingly a gRNA targeting IRF6 was enriched suggesting it normally represses self-renewal. SOX9 knockdown led to DE of about 455 genes. As expected SOX9 suppresses premature differentiation of distal epithelial progenitors into airway epithelial cell types but also differentiation into other foregut lineages. SOX9 also suppresses metabolic processes and promotes cell division. SOX9 targeted DamId was used to identify direct SOX9 target genes. 171 direct SOX9 target genes were identified. Interestingly SOX9 regulates Etv4/5 but also several Wnt pathway genes. SOX9 was found to be a direct Wnt target gene but also further enhanced Wnt signaling in a feedforward loop. Etv4/5 double knockdown like SOX9 knockdown negatively affected progenitor renewal. Removal of RTK ligands (EGF, FGF7 and FGF10) did not seem to affect SOX9 expression at least in the presence of other Wnt activators in the culture medium. TaDa analysis of Etv4/5 suggests that Etv4/5 and SOX9 may coregulate certain target genes though through binding at different loci.

Major concerns.
A) In the mouse Fgf10 has been shown to induce Sox9 expression and inhibition of Fgf10 signaling also suppresses Sox9 expression. It is thought that Fgf10 can does this via PI3K AKT signaling to inhibit GSK3β. Could it be that R-spondin-1 and CHIR99021 levels in the culture medium are so high that they compensate for RTK ligand loss? Also 100ng/ml of Fgf10 is usually not sufficient to induce proper Fgf10 signaling.
To test this the authors should try
1) Increase FGF10 to 500ng/ml and probe for Sox9 expression.
2) Increase FGF10 to 500ng/ml and remove or reduce R-spondin-1 and/or CHIR99021 (especially the GSK3β inhibitor CHIR99021).

We have now performed the exact experiments suggested by the reviewer. These are shown in Fig. EV5A. In the absence of WNT activators, or with reduced WNT activators, for 6 days increased FGF10 is not sufficient to rescue *SOX9* transcription. We conclude that in the context of this 6 day short-term experiment, WNT signalling is necessary to promote SOX9 transcription and RTK signalling is not.

Regulation of SOX9 by FGF signalling is likely to complex in the developing human lungs. For example, the Al Alam lab found that treatment of human lung explants with FGF7, or FGF9, or FGF10 resulted in a smaller number of SOX9+ lung bud tip progenitors (Danopoulos et al., 2019; https://doi.org/10.1002/path.5188).

B) The authors perform a pretty good analysis of which genes are directly and indirectly regulated by SOX9 but fail to do so for Etv4/5. Which genes are differentially expressed in the Etv4/5 double knockdown and what are the direct Etv4/5 target genes and which genes

are likely co-regulated by Etv4/5 and Sox9?

As suggested by the reviewer, we have now performed the RNA sequencing analysis of the *ETV4/5* double knock-down organoids and integrated this with the ETV5 binding data (Figure EV5E-F). We were surprised to find that there were only 42 overlapping differentially expressed genes (DEGs) between the 2 organoid lines (Fig. EV5E) and that these mostly affected cell proliferation. Only 26 of the DEGs were also directly bound by ETV5, and only 6 were also SOX9 direct targets. This overall small number of DEGs partially reflects the biological variability in levels of baseline gene expression between organoids (which is illustrated clearly by comparing the non-targeting controls in Fig. EV5E). However, more stringent experimental cut-offs and the same line-to-line biological variability in transcription levels did not prevent knock-down of *SOX9* having a significant effect on transcription (Figs. 2G, EV3E). We note that two other studies have found binding of ETV5 to many more genes than were actually differentially expressed (>10,000 binding sites and 185 DEGs in adult mouse alveolar type 2 cells; 754 binding sites and 85 DEGs in mouse ESCs), suggesting that this could be a feature of ETV proteins Zhang et al., 2017, https://doi.org/10.1073/pnas.1621177114; Kalkan et al., 2019, https://doi.org/10.1016/j.stem.2019.03.017).

We therefore interpret our data overall as meaning that SOX9 is a key upstream regulator of the self-renewal transcriptional network in lung bud tip progenitors. Whereas, ETV4 and 5 directly promote cell proliferation, but work in combination with other TFs at their other genomic binding sites. This is now explored in the Discussion section, particularly in the context of a combinatorial TF network regulating self-renewal of the human lung bud tip progenitors and the possibility of further combining CRISPR screens and DNA-binding analysis to explore this in the future.

Minor concerns

C) Which genes are differentially expressed in the IRF6 knockdown and what are the direct IRF6 target genes?

Although IRF6 is potentially interesting, we made the decision not to focus on IRF6 in this short manuscript and therefore this point is out of scope.

1st Revision - Editorial Decision 28th Jul 2022

Thank you for submitting a revised version of your manuscript. Your study has now been seen by all original referees, who find that their previous concerns have been addressed and recommend publication of the manuscript. There remain only a couple of editorial issues that have to be solved before I can extend formal acceptance of the manuscrip.:

Please let me know if you have any further questions regarding any of these points. You can use the link below to upload the revised files.

Thank you again for giving us the chance to consider your manuscript for The EMBO Journal. I look forward to receiving the final version.

Referee #1:

The revised manuscript addressed all my previous concerns. I have no further comments.

Referee #2:

The authors adequately addressed my comments.

Referee #3:

The authors have addressed all my concerns.

The authors performed the requested editorial changes.

2nd Revision - Editorial Decision 16th Aug 2022

Editor accepted the manuscript.

EMBO Press Author Checklist

Corresponding Author Name: Emma Rawlins	
Journal Submitted to: EMBO Journal	
Manuscript Number: EMBOJ-2022-111338R	

USEFUL LINKS FOR COMPLETING THIS FORM
The EMBO Journal - Author Guidelines
EMBO Reports - Author Guidelines
Molecular Systems Biology - Author Guidelines
EMBO Molecular Medicine - Author Guidelines

Reporting Checklist for Life Science Articles (updated January 2022)
This checklist is adapted from Materials Design Analysis Reporting (MDAR) Checklist for Authors. MDAR establishes a minimum set of requirements in transparent reporting in the life sciences (see Statement of Task: 10.31222/osf.io/9sm4x). Please follow the journal's guidelines in preparing your manuscript.
Please note that a copy of this checklist will be published alongside your article.

Abridged guidelines for figures
1. Data
The data shown in figures should satisfy the following conditions:
- the data were obtained and processed according to the field's best practice and are presented to reflect the results of the experiments in an accurate and unbiased manner.
- ideally, figure panels should include only measurements that are directly comparable to each other and obtained with the same assay.
- plots include clearly labeled error bars for independent experiments and sample sizes. Unless justified, error bars should not be shown for technical replicates.
- if n<5, the individual data points from each experiment should be plotted. Any statistical test employed should be justified.
- Source Data should be included to report the data underlying figures according to the guidelines set out in the authorship guidelines on Data Presentation.

2. Captions
Each figure caption should contain the following information, for each panel where they are relevant:
- a specification of the experimental system investigated (eg cell line, species name).
- the assay(s) and method(s) used to carry out the reported observations and measurements.
- an explicit mention of the biological and chemical entity(ies) that are being measured.
- an explicit mention of the biological and chemical entity(ies) that are altered/varied/perturbed in a controlled manner.
- the exact sample size (n) for each experimental group/condition, given as a number, not a range;
- a description of the sample collection allowing the reader to understand whether the samples represent technical or biological replicates (including how many animals, litters, cultures, etc.).
- a statement of how many times the experiment shown was independently replicated in the laboratory.
- definitions of statistical methods and measures:
  - common tests, such as t-test (please specify whether paired vs. unpaired), simple χ2 tests, Wilcoxon and Mann-Whitney tests, can be unambiguously identified by name only, but more complex techniques should be described in the methods section;
  - are tests one-sided or two-sided?
  - are there adjustments for multiple comparisons?
  - exact statistical test results, e.g., P values = x but not P values < x;
  - definition of 'center values' as median or average;
  - definition of error bars as s.d. or s.e.m.

Please complete ALL of the questions below.
Select "Not Applicable" only when the requested information is not relevant for your study.

Materials

Newly Created Materials	Information included in the manuscript?	In which section is the information available? (Reagents and Tools Table, Materials and Methods, Figures, Data Availability Section)
New materials and reagents need to be available; do any restrictions apply?	Yes	Materials and methods/data availability section

Antibodies	Information included in the manuscript?	In which section is the information available? (Reagents and Tools Table, Materials and Methods, Figures, Data Availability Section)
For **antibodies** provide the following information:<br>- Commercial antibodies: RRID (if possible) or supplier name, catalogue number and or/clone number<br>- Non-commercial: RRID or citation	Yes	Materials and methods

DNA and RNA sequences	Information included in the manuscript?	In which section is the information available? (Reagents and Tools Table, Materials and Methods, Figures, Data Availability Section)
**Short novel DNA or RNA including primers, probes**: provide the sequences.	Yes	Materials and methods (EV Dataset 8)

Cell materials	Information included in the manuscript?	In which section is the information available? (Reagents and Tools Table, Materials and Methods, Figures, Data Availability Section)
**Cell lines:** Provide species information, strain. Provide accession number in repository **OR** supplier name, catalog number, clone number, and/**OR** RRID.	Not Applicable	
**Primary cultures:** Provide species, strain, sex of origin, genetic modification status.	Yes	Materials and methods
Report if the cell lines were recently **authenticated** (e.g., by STR profiling) and tested for mycoplasma contamination.	Not Applicable	

Experimental animals	Information included in the manuscript?	In which section is the information available? (Reagents and Tools Table, Materials and Methods, Figures, Data Availability Section)
**Laboratory animals or Model organisms:** Provide species, strain, sex, age, genetic modification status. Provide accession number in repository **OR** supplier name, catalog number, clone number, **OR** RRID.	Not Applicable	
**Animal observed in or captured from the field:** Provide species, sex, and age where possible.	Not Applicable	
Please detail **housing and husbandry conditions**.	Not Applicable	

Plants and microbes	Information included in the manuscript?	In which section is the information available? (Reagents and Tools Table, Materials and Methods, Figures, Data Availability Section)
**Plants:** provide species and strain, ecotype and cultivar where relevant, unique accession number if available, and source (including location for collected wild specimens).	Not Applicable	
**Microbes:** provide species and strain, unique accession number if available, and source.	Not Applicable	

Human research participants	Information included in the manuscript?	In which section is the information available? (Reagents and Tools Table, Materials and Methods, Figures, Data Availability Section)
If collected and within the bounds of privacy constraints report on age, sex and gender or ethnicity for all study participants.	Not Applicable	

Core facilities	Information included in the manuscript?	In which section is the information available? (Reagents and Tools Table, Materials and Methods, Figures, Data Availability Section)
If your work benefited from core facilities, was their service mentioned in the acknowledgments section?	Yes	Acknowledgements section

Design

Study protocol	Information included in the manuscript?	In which section is the information available? (Reagents and Tools Table, Materials and Methods, Figures, Data Availability Section)
If study protocol has been **pre-registered, provide DOI in the manuscript**. For clinical trials, provide the trial registration number **OR** cite DOI.	Not Applicable	
Report the **clinical trial registration number** (at ClinicalTrials.gov or equivalent), where applicable.	Not Applicable	

Laboratory protocol	Information included in the manuscript?	In which section is the information available? (Reagents and Tools Table, Materials and Methods, Figures, Data Availability Section)
Provide DOI OR other citation details if **external detailed step-by-step protocols** are available.	Not Applicable	

Experimental study design and statistics	Information included in the manuscript?	In which section is the information available? (Reagents and Tools Table, Materials and Methods, Figures, Data Availability Section)
Include a statement about **sample size** estimate even if no statistical methods were used.	Yes	Figure legends
Were any steps taken to minimize the effects of subjective bias when allocating animals/samples to treatment (e.g. **randomization procedure**)? If yes, have they been described?	Not Applicable	
Include a statement about **blinding** even if no blinding was done.	Yes	Materials and methods
Describe **inclusion/exclusion criteria** if samples or animals were excluded from the analysis. Were the criteria pre-established?<br><br>If sample or data points were omitted from analysis, report if this was due to attrition or intentional exclusion and provide justification.	Not Applicable	All data was included
For every figure, are **statistical tests** justified as appropriate? Do the data meet the assumptions of the tests (e.g., normal distribution)? Describe any methods used to assess it. Is there an estimate of variation within each group of data? Is the variance similar between the groups that are being statistically compared?	Yes	Statistical tests described in the figure legends. An estimate of variation was not performed.

Sample definition and in-laboratory replication	Information included in the manuscript?	In which section is the information available? (Reagents and Tools Table, Materials and Methods, Figures, Data Availability Section)
In the figure legends: state number of times the experiment was **replicated** in laboratory.	Yes	Figure legends
In the figure legends: define whether data describe **technical or biological replicates**.	Yes	Figure legends

Ethics

Ethics	Information included in the manuscript?	In which section is the information available? (Reagents and Tools Table, Materials and Methods, Figures, Data Availability Section)
Studies involving **human participants**: State details of **authority granting ethics approval** (IRB or equivalent committee(s), provide reference number for approval.	Yes	Materials and methods
Studies involving **human participants**: Include a statement confirming that **informed consent** was obtained from all subjects and that the experiments conformed to the principles set out in the WMA Declaration of Helsinki and the Department of Health and Human Services Belmont Report.	Not Applicable	
Studies involving **human participants:** For publication of **patient photos**, include a statement confirming that consent to publish was obtained.	Not Applicable	
Studies involving experimental **animals**: State details of **authority granting ethics approva**l (IRB or equivalent committee(s), provide reference number for approval. Include a statement of compliance with ethical regulations.	Not Applicable	
Studies involving **specimen and field samples:** State if relevant **permits** obtained, provide details of authority approving study; if none were required, explain why.	Not Applicable	

Dual Use Research of Concern (DURC)	Information included in the manuscript?	In which section is the information available? (Reagents and Tools Table, Materials and Methods, Figures, Data Availability Section)
Could your study fall under dual use research restrictions? Please check biosecurity documents and list of **select agents and toxins** (CDC): https://www.selectagents.gov/sat/list.htm	Not Applicable	
If you used a select agent, is the security level of the lab appropriate and reported in the manuscript?	Not Applicable	
If a study is subject to dual use research of concern regulations, is the name of the **authority granting approval and reference number** for the regulatory approval provided in the manuscript?	Not Applicable	

Reporting

The MDAR framework recommends adoption of discipline-specific guidelines, established and endorsed through community initiatives. Journals have their own policy about requiring specific guidelines and recommendations to complement MDAR.

Adherence to community standards	Information included in the manuscript?	In which section is the information available? (Reagents and Tools Table, Materials and Methods, Figures, Data Availability Section)
State if relevant guidelines or checklists (e.g., **ICMJE, MIBBI, ARRIVE, PRISMA**) have been followed or provided.	Not Applicable	
For **tumor marker prognostic studies,** we recommend that you follow the **REMARK** reporting guidelines (see link list at top right). See author guidelines, under 'Reporting Guidelines'. Please confirm you have followed these guidelines.	Not Applicable	
For **phase II and III randomized controlled trials**, please refer to the **CONSORT** flow diagram (see link list at top right) and submit the CONSORT checklist (see link list at top right) with your submission. See author guidelines, under 'Reporting Guidelines'. Please confirm you have submitted this list.	Not Applicable	

Data Availability

Data availability	Information included in the manuscript?	In which section is the information available? (Reagents and Tools Table, Materials and Methods, Figures, Data Availability Section)
Have **primary datasets** been deposited according to the journal's guidelines (see 'Data Deposition' section) and the respective accession numbers provided in the Data Availability Section?	Yes	Data availability section in Material and Methods
Were **human clinical and genomic datasets** deposited in a public access-controlled repository in accordance to ethical obligations to the patients and to the applicable consent agreement?	Not Applicable	
Are **computational models** that are central and integral to a study available without restrictions in a machine-readable form? Were the relevant accession numbers or links provided?	Not Applicable	
If publicly available data were reused, provide the respective **data citations in the reference list**.	Not Applicable	
